# Skeletal Effects of Slow Maxillary Expansion in Mixed Dentition Stage: A Systematic Review and Meta‐Analysis

**DOI:** 10.1002/cre2.70488

**Published:** 2026-11-09

**Authors:** Suzan Ahmad Ibraheem, Tuqa Raghis, Mawia Karkoutly, Mohamed Youssef

**Affiliations:** ^1^ Department of Orthodontics, Faculty of Dentistry Damascus University Damascus Syrian Arab Republic; ^2^ Department of Pediatric Dentistry, Faculty of Dentistry Damascus University Damascus Syrian Arab Republic

**Keywords:** quad–helix, removable expansion plate, skeletal changes

## Abstract

**Objectives:**

Many cases of maxillary arch expansion require skeletal improvement of varying degrees. Therefore, the current systematic review and meta‐analysis aim to evaluate the effectiveness of the most widespread slow maxillary expansion (SME) in achieving the necessary skeletal changes during the mixed dentition stage in addition to dentoalveolar ones.

**Material and Methods:**

An electronic search of five databases (PubMed, Scopus, Embase, Cochrane Library, and Google Scholar) was followed by manual searches in several journals. Both randomized controlled trials (RCTs) and nonrandomized studies were included, while case reports were excluded. Risk of bias was assessed using the Revised Version of Cochrane Risk‐Of‐Bias (ROB 2.0) tool for randomized studies (high, low, or some concerns) for individual elements from five domains (selection, intervention, missing data, outcome measurement, and reporting) and the ROBINS‐I tool for nonrandomized studies. The certainty of the evidence was evaluated according to the Grading of Recommendations Assessment, Development and Evaluation (GRADE) approach.

**Results:**

In total, 108 full texts from 1427 identified articles were screened; 28 studies were included in this review (7 RCTs and 21 non‐RCTs), and 11 were appropriate for quantitative synthesis studies. Outcomes assessed dental and skeletal changes, such as inter‐molar/inter‐canine widths, as well as maxillary width. The study showed different changes related to the expansion device. Whereas, the meta‐analysis revealed that a greater increase in the inter‐molar width was found in the quad–helix (QH) group than in the removable plate (RP) group, and the difference was statistically significant, while the difference was not significant in inter‐canine width. Also, the increase in maxillary width was significant by using QH and RP. Thus, the QH was more effective than the expansion plate.

**Conclusions:**

It can be concluded that SME during the mixed dentition can lead to different amounts of skeletal changes in addition to the dentoalveolar changes, and this amount is related to the expansion appliance used. The main limitation is that few studies have measured changes in maxillary width.

## 1 Introduction

Maxillary transverse deficiency, also known as maxillary hypoplasia, represents a significant clinical challenge that adversely affects both facial development and dentoalveolar integrity (McNamaraa 2000; Consolaro and Consolaro 2018). Early intervention is crucial to restore proper transverse skeletal relationships between the jaw bases, as this forms the foundation for achieving optimal occlusal stability and function (McNamaraa 2000; Consolaro and Consolaro 2018).

Clinical assessment of transverse maxillary deficiency (TMD) involves evaluation of multiple features, including maxillary arch form and symmetry, palatal vault morphology, occlusal relationships, breathing patterns (oral vs. nasal), and buccal corridor dimensions during smiling (Marshall et al. 2005). The condition typically presents with characteristic manifestations such as dental crowding, tooth rotations, buccal/palatal displacement of teeth, high‐arched palate, and V‐shaped dental arches. Notably, mouth breathing has been identified as a contributing etiological factor for TMD development (Marshall et al. 2005). TMD most commonly manifests as a posterior crossbite, which can be unilateral or bilateral, and can range from complete to partial involvement. Interestingly, some patients may exhibit TMD without crossbite when concurrent mandibular arch constriction exists. Epidemiological studies report a 7.7% prevalence of posterior crossbite in deciduous and mixed dentition populations, with incidence rates increasing through adulthood (Brunelle et al. 1996; Malandris and Mahoney 2004). Furthermore, TMD often coexists with anteroposterior discrepancies and can be categorized as either absolute (real) or compensatory (relative) (Karad 2014). Diagnostic confirmation and quantification of the skeletal involvement can be achieved through frontal cephalometric analysis using established methods such as those described by Ricketts, Sassouni, Grummons, or Reynecke (Bouletreau and Raberin 2016).

Posterior crossbite requires active intervention as it does not resolve spontaneously (da Silva Filho et al. 1989). The primary treatment objective involves maxillary arch expansion to restore proper transverse dimensions. This correction is essential for normalizing temporomandibular joint (TMJ) function and achieving unimpeded mandibular closure (Myers et al. 1980; Harberson and Myers 1978). Without treatment, persistent crossbite may induce chronic muscular and TMJ dysfunction, potentially progressing to significant facial asymmetries that could necessitate surgical intervention in later years (Agostino et al. 2014). Slow maxillary expansion (SME) devices achieve the required expansion through controlled low‐magnitude forces of approximately 2 pounds, promoting physiological skeletal adaptation (Hicks 1978).

Existing research has examined various aspects of maxillary expansion therapy. Previous systematic reviews have investigated early intervention for unilateral posterior crossbite in primary and early mixed dentition (Petrén et al. 2003; Caroccia et al. 2020). Zuccati et al. (2013) analyzed randomized controlled trials (RCTs) from the past decade comparing rapid (RME), slow (SME), and semi‐rapid maxillary expansion (SRME) across different age groups. While some studies have evaluated posterior crossbite treatment outcomes broadly (Ugolini et al. 2021), others, like Lagravère et al. (2005), focused specifically on short‐term SME effects, albeit without control group comparisons. Recent comprehensive analyses include a 2021 umbrella review comparing RME and SME craniofacial outcomes (Aboalnaga 2021) and a meta‐analysis by Bucci et al.'s (2016) of fixed appliance therapies. The most current 2023 systematic review expanded this comparison across various age groups (Inchingolo et al. 2023).

This systematic review and meta‐analysis aimed to evaluate the skeletal and dental effects of SME in mixed dentition, with a focus on comparing different expansion appliances, quantifying transverse dimensional changes, and assessing the clinical implications of treatment protocols. It aimed to determine the efficacy of SME in achieving maxillary expansion, compare outcomes between fixed and removable appliances, and identify gaps in the existing evidence regarding skeletal versus dentoalveolar changes. To our knowledge, no systematic review has assessed both skeletal and dental changes resulting from the SME in mixed dentition; therefore, this systematic review was conducted.

## 2 Materials and Methods

### 2.1 Study Design

The systematic review was conducted according to the guidelines of the Cochrane Handbook for Systematic Reviews of Interventions, 2nd edition (Cumpston et al. 2019) and the Preferred Reporting Items for Systematic Reviews and Meta‐Analyses (PRISMA) (Liberati et al. 2009). It was registered with PROSPERO number ID = CRD42023487507 (12/12/2023).

### 2.2 Inclusion Criteria

According to the population, intervention, comparison, outcomes, and study design (PICOS) framework, patients with maxillary arch constriction in the mixed dentition period were chosen as the targeted population. Concerning the “intervention,” interceptive treatment by SME using fixed or removable appliances was accepted for inclusion. The “comparison” group was constructed from scheduled patients who received any expansion appliance, non‐treated patients, or a normal control group. The “outcomes” of interest were inter‐molar (IM) and inter‐canine (IC) widths, and maxillary width (MW). In addition to the activated protocol and treatment duration. RCTs and nonrandomized studies were included.

### 2.3 Exclusion Criteria

Case reports or case series, editorials, personal opinions, reviews, and technique description articles without reported samples.

Studies included RME and patients with Class III malocclusion or cleft lip and/or palate.

### 2.4 Search Strategy

The search strategy included a review of English electronic databases supplemented by a manual search. The electronic search was carried out by an independent reviewer (S.A.I.) and was performed in PubMed, the Cochrane Library, Scopus, EMBASE, and Google Scholar. The electronic search was carried out in the following journals: the *American Journal of Orthodontics and Dentofacial Orthopedics*, the *European Journal of Orthodontics*, the *Angle Orthodontist*, and the *International Journal of Orthodontics*. The studies published via advanced searches were searched with no restrictions on year or country of origin. The last date of search was on 01/04/2025. The keywords used and the details of the search strategy are provided in Table 1.

**Table 1:** Electronic search strategy.

Database	Search strategy	Outcomes
**PubMed** http://www.ncbi.nlm.nih.gov/pubmed/ All fields	**#1** (“slow maxillary expansion” OR “slow maxilla expansion” OR “dental expansion”) **#2 (“young” OR “Preadolescent” OR “children” OR “mixed dentition”)** **#3 (“removable expansion appliance” OR “removable plate” OR “quad–Helix”)** **#4 (“transverse deficiency” OR “transverse constriction” OR “posterior cross bite”** OR “lateral cross bite”) **#5 #1 AND #2 AND #3 AND #4**	175
**Cochrane Library** http://www.cochranelibrary.com/ TITLE‐ABS‐KEY	#1 (“slow maxillary expansion” OR “slow maxilla expansion” OR “dental expansion”) **#2 (“young” OR “Preadolescent” OR “children” OR “mixed dentition”)** **#3 (“removable expansion appliance” OR “removable plate” OR “quad–Helix”)** **#4 (“transverse deficiency” OR “transverse constriction” OR “posterior cross bite”** OR “lateral cross bite”) **#5 #1 AND #2 AND #3 AND #4**	36
**EMBASE** https://www.embase.com/	**#1** (“slow maxillary expansion” OR “slow maxilla expansion” OR “dental expansion”) **#2 (“young” OR “Preadolescent” OR “children” OR “mixed dentition”)** **#3 (“removable expansion appliance” OR “removable plate” OR “quad–Helix”)** **#4 (“transverse deficiency” OR “transverse constriction” OR “posterior cross bite”** OR “lateral cross bite”) **#5 #1 AND #2 AND #3 AND #4**	15
**Google Scholar** https://scholar.google.com/	**#1** (“slow maxillary expansion” OR “slow maxilla expansion” OR “dental expansion”) **#2 (“young” OR “Preadolescent” OR “children” OR “mixed dentition”)** **#3 (“removable expansion appliance” OR “removable plate” OR “quad–Helix”)** **#4 (“transverse deficiency” OR “transverse constriction” OR “posterior cross bite”** OR “ lateral cross bite”) **#5 #1 AND #2 AND #3 AND #4**	636
**Scopus** http://www.scopus.com/	**#1** TITLE‐ABS‐KEY (“slow maxillary expansion” OR “slow maxilla expansion” OR “dental expansion”) **#2** TITLE‐ABS‐KEY **(“young” OR “Preadolescent” OR “children” OR “mixed dentition”)** **#3** TITLE‐ABS‐KEY **(“removable expansion appliance” OR “removable plate” OR “quad–Helix”)** **#4** TITLE‐ABS‐KEY **(“transverse deficiency” OR “transverse constriction” OR “posterior cross bite”** OR “lateral cross bite”) **#5 #1 AND #2 AND #3 AND #4**	167
***The Angle Orthodontist*** https://meridian.allenpress.com/angle-orthodontist/	**“slow maxillary expansion”**	102
***European Journal of Orthodontics*** https://academic.oup.com/ejo/	**“slow maxillary expansion”**	150
***International journal of Orthodontics*** https://www.iaortho.org/	**“slow maxillary expansion”**	27
https://www.sciencedirect.com/journal/american-journal-of-orthodontics-and-dentofacial-orthopedics	**“slow maxillary expansion”**	119
**Overall**		1427

#1 (“slow maxillary expansion” OR “slow maxilla expansion” OR “dental expansion”);

#2 (“young” OR “Preadolescent” OR “children” OR “mixed dentition”);

#3 (“removable plate” OR “removable expansion appliances” OR “Quad–Helix”);

#4 (“transversal deficiency” OR “transversal constriction” OR “posterior crossbite” OR “lateral cross bite”); and

#5 #1 AND #2 AND #3 AND #4.

### 2.5 Study Selection and Data Extraction

The selection of the studies was carried out independently by two reviewers (S.A.I., T.R.) in accordance with eligibility criteria, and disagreements were resolved by asking a third author (M.Y.). First, all titles were screened, and the irrelevant articles were excluded. Then, the remaining articles were evaluated to eliminate studies based on data obtained from abstracts. Finally, the full text of the remaining articles was evaluated to confirm acceptability according to the eligibility criteria. Data were extracted independently from the included studies according to the PICOS question and arranged in fields; general information (author name and publication date), study design, participants (size, age, and sex of samples), intervention (type of expansion/appliance, expansion protocol, and the treatment duration), outcomes (primary outcomes; IC, IM, and MW/methods of measurement), and study main findings.

### 2.6 Assessment of Risk of Bias in Individual Studies

Two reviewers (S.A.I. and T.R.) independently assessed the quality of the included studies, and due to a lack of consistency, a third author (M.Y.) was consulted to resolve the issue. The reviewers used the Revised Version of Cochrane Risk‐Of‐Bias (ROB 2.0) tool for randomized studies (high, low, or some concerns) for individual elements of five domains (selection, intervention, missing data, outcome measurement, and reporting) (Sterne et al. 2019). The overall risk of bias of the individual studies was evaluated. A high risk of bias was considered when one or more fields were assessed as having a high risk of bias, some concerns risk of bias when one or more fields were assessed as having some concerns risk of bias, but not judged as “High Risk” in any domain, and low risk of bias when all fields were assessed as having a low risk of bias.

The Risk‐Of‐Bias in Nonrandomized Studies of Intervention (ROBINS‐I) tool for nonrandomized studies (Sterne et al. 2016; Thomson et al. 2018), to evaluate potential sources of bias across the seven ROBINS‐I domains (Supporting Material 1). The Risk‐Of‐Bias Visualization tool (robvis) was used to distinguish the risk of bias assessment (McGuinness and Higgins 2021).

Studies were excluded from the quantitative analysis if they were rated as at critical risk of bias or serious risk of bias and were considered “poor quality” after the NIH assessment. Since uncontrolled (one‐group) before‐and‐after revisions are usually considered to be at serious or critical risk of bias according to the ROBINS‐I tool, an additional assessment was applied to include them anyway (Ma et al. 2020), knowing that they could provide an answer to the PICOS question, using the National Institutes of Health (NIH) quality assessment tool for before‐and‐after studies (Pre, Post) without a control group (Health NIo 2021). These studies were excluded from the quantitative analysis if they were considered “poor quality” after the NIH assessment.

### 2.7 Data Synthesis, Assessment of Risk of Bias Across Studies, and Assessment of the Strength of Evidence

Meta‐analysis was done using Review Manager Software, Version 5.4.1 (Copenhagen; The Nordic Cochrane Centre, Cochrane Collaboration). The mean difference was used as a summary statistic, and a random‐effect method was used for meta‐analysis. The level of significance was set at 0.05. Heterogeneity was first evaluated clinically according to appliance type and baseline characteristics of participants. *I* ^2^ and chi‐square tests were performed to quantify the heterogeneity across studies, and their values were explained as follows: low heterogeneity: 0%–40%, moderate heterogeneity: 30%–60%, substantial/high heterogeneity: 50%–90%, and considerable/very high heterogeneity: 75%–100% (Higgins et al. 2003).

## 3 Results

### 3.1 The Flow of the Search Strategy

In total, 398 English articles were obtained by hand searching the reference lists of the selected articles, and 1029 articles were collected from the electronic literature search in PubMed, Scopus, the Cochrane Library, EMBASE, and Google Scholar. In addition, duplicate articles were removed, and 108 articles were checked. A total of 108 articles were excluded after the full‐text assessment. A total of 28 studies were included; 11 of them were included in the quantitative synthesis (meta‐analysis). Figure 1 shows the PRISMA flow diagram.

**Figure 1:**
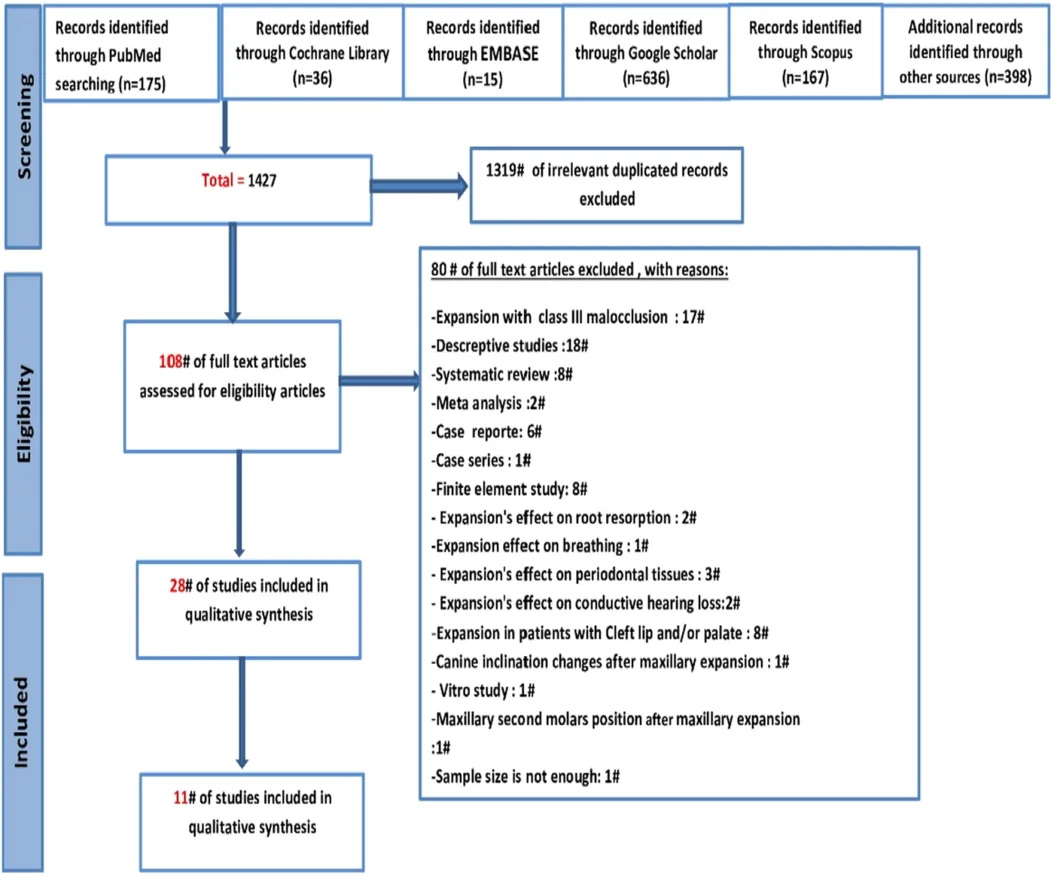
PRISMA flow chart of the study selection process.

### 3.2 Characteristics of the Included Studies

The studies included in this systematic review were 28 studies (7 RCTs and 21 studies were nonrandomized studies).

The slow expansion groups in the studies involved 1204 patients, and the main inclusion criteria were as follows: (1) maxillary arch constriction with or without a lateral crossbite, (2) mixed dentition, and (3) fully erupted upper and lower first molars.

Fourteen of the included studies used quad–helix (QH) (involving 384 patients) (Godoy et al. 2011; Frank and Engel 1982; Bell and LeCompte 1981; Boysen et al. 1992; Ladner and Muhl 1995; Sandikçiolu and Hazar 1997; Erdinç et al. 1999; Vargo et al. 2007; Petrén and Bondemark 2008; Huynh et al. 2009; Wong et al. 2011; Corbridge et al. 2011; Shundo et al. 2012; Shoaib et al. 2017), seven of the included studies (involving 371 patients) used removable expansion plates (Godoy et al. 2011; Boysen et al. 1992; Erdinç et al. 1999; Petrén and Bondemark 2008; Defraia et al. 2007; Van de Velde et al. 2021; Zhang et al. 2023), two studies used Minne expanders (involving 22 patients) (Mossaz‐Joëlson and Mossaz 1989; Akkaya 1998), three studies used a Ni‐Titanium Palatal Expander (NPE) (involving 33 patients) (Ciambotti et al. 2001; Ferrario 2003; Nagrik and Bhad 2014), and six studies used Hass and Hyrax expanders (involving 256 patients) (Vargo et al. 2007; Huynh et al. 2009; Wong et al. 2011; Martina et al. 2012; Brunetto et al. 2013; Bukhari et al. 2018). Two studies used composites only or the TransForce transverse appliance (TTA) (involving 25 patients) (Petrén and Bondemark 2008; Nagrik and Bhad 2014). Seven studies included control groups of untreated subjects (Godoy et al. 2011; Erdinç et al. 1999; Petrén and Bondemark 2008; Wong et al. 2011; Shundo et al. 2012; Defraia et al. 2007; Ferrario 2003), normal occlusion subjects (Bukhari et al. 2018), or RME patients (Cossellu et al. 2020). Four studies compared the QH and RP (Boysen et al. 1992; Erdinç et al. 1999; Petrén and Bondemark 2008; Wong et al. 2011).

The outcome measurements included frontal cephalograms (1 study; Defraia et al. 2007), cone beam computed tomography (CBCT) images (4 studies; Corbridge et al. 2011; Zhang et al. 2023; Brunetto et al. 2013; Serafin et al. 2022), and computed tomography (CT) images (Martina et al. 2012). The cast models were evaluated in fifteen studies (Godoy et al. 2011; Bell and LeCompte 1981; Ladner and Muhl 1995; Vargo et al. 2007; Petrén and Bondemark 2008; Huynh et al. 2009; Wong et al. 2011; Shundo et al. 2012; Van de Velde et al. 2021; Akkaya 1998; Ciambotti et al. 2001; Ferrario 2003; Nagrik and Bhad 2014; Bukhari et al. 2018; Cossellu et al. 2020), and both the cast models and frontal cephalograms were evaluated in seven studies (Frank and Engel 1982; Boysen et al. 1992; Sandikçiolu and Hazar 1997; Erdinç et al. 1999; Shoaib et al. 2017; Mossaz‐Joëlson and Mossaz 1989; Paoloni et al. 2022). Details are provided in Table 2.

**Table 2:** Characteristics of the included trials.

Ratio	Expansion obtained on the maxillary arch	Treatment time/activated protocol	Participants/method	Study
MW/IM = 52.67%	IC: 3.56 ± 1.99^a^ IM: 3.55 ± 1.61 mm^a^ MW: 1.87 ± 1.24 mm^a^	‐ Treatment duration (9.09 ± 4.94 months) ‐ The expansion screw was activated at 90° a week	44 patients (7.75 ± 1.10 y) RP CBCT	Zhang et al. (2023) A retrospective study
MW/IM = 59%	SME: IM: 2.2 ± 1.7^a^ MW: 1.3 ± 1.3^a^ RME: IM: 3.9 ± 1.9^a^ MW: 1.3 ± 1.8^a^	‐ Treatment duration/activated protocol ‐ SME: 3 months/1 mm per month ‐ RME: 2 months/0.25 mm per day	32 patients ‐ SME: 16 (5 m/11 f) (8.96 ± 0.92) LE expander ‐ RME: 16 (6 m/10 f) (8.50 ± 1.39 y) Hyrax expander CBCT	Serafin et al. (2022) RCT
MW/IM = 69.69%	Leaf EP: IC: 2.2 ± 1.4 IM: 3.3 ± 1.9^a^ MW: 2.3 ± 1.6^a^ RME: IC: 3.0 ± 1.3 IM: 3.2 ± 1.6 MW: 3.7 ± 2.4	‐ Leaf Expander (6–6.5 months) 1.5 mm monthly. An initial expansion of 4.5 mm was achieved in about 2–3 months ‐ Conventional RME screw (1.5–2 months)	56 patients (early or intermediate mixed dentition stage ‐ 28 Leaf Expander ‐ 28 conventional RME screw PA cephalograms and dental casts	Paoloni et al. (2022) RCT
—	IC: 3.8 ± 1.8^a^ IM: 3.5 ± 2.1^a^	‐ Treatment duration 6.9 months 0.25 mm per week‐	226 patients (101 m/125 f) (8.7 ± SD 1.0 years) ‐ RP ‐ Dental casts	Van de Velde et al. (2021) A prospective cohort study
—	‐ SME: IC: 5.22 ± 1.91^a^ IM: 2.51 ± 1.58^a^ ‐ RME: IC: 3.63 ± 1.51^a^ IM: 3.81 ± 1.43^a^	‐ Active phase and retention of 9–11 months ‐ SME: Screw activation was done 1.5 mm monthly ‐ CG: RME: Screw activation was done three times per day for 10 days	‐ 90 patients (43 m/47 f) (7.5 ± 1.5 years) ‐ SME: Leaf Expander: 69 patients (33 m/36 f) (7.6 ± 1.7 y) ‐ CG: RME: Haas Expander: 21 patients (10 m/11 f) (7.4 ± 1.2 y) ‐ Dental casts	Cossellu et al. (2020) A retrospective case‐control study
—	‐ TG: IC: 4.65 ± 2.1^a^ IM: 4.76 ± 2.91^a^ CG: IC: 0.57 ± 2.1‐ IM: 0.47 ± 2.3	‐ Records were taken after 1 year ‐ Expansion protocol was one quarter‐turn every 2 days until overcorrection	60 patients ‐ TG: Haas expander (11 m/19 f) (8.36 ± 6 0.7 y) ‐ CG: (11 m/19 f) (8.30 ± 0.75) ‐ Dental casts	Bukhari et al. (2018) A retrospective case‐control study
MW/IM = 14.15%	IM: 10.10 ± 4.88 mm^a^ MW: 1.43 ± 1.01 mm^a^	Treatment duration‐ (3–4 months) ‐ New activations were posted on 40–40‐day period	14 patients (6–12 y) QH ‐ PA cephalograms and dental cast	Shoaib et al. (2017) A prospective study
MW/IM = 16%–20%	‐ TTA: IC: 3.59 ± 1.25^a^ IM: 4.99 ± 1.68^a^ MW: 0.64 ± 0.55^a^ NPE: IC: 2.57 ± 1.15^a^ IM: 4.88 ± 2.06^a^ MW: 0.84 ± 0.17^a^	Expansion stopped when the lingual cusp of the maxillary permanent first molars or the primary second molars contacted the opposing mandibular buccal cusp	20 patients (9.5–13 y) ‐ TTA: 10 patients ‐ NPE: 10 patients Dental casts	Nagrik and Bhad (2014) RCT
—	‐ SME: IM: 9.02 ± 1.70^a^ ‐ RME: IM: 9.26 ± 2.05^a^	‐ Records were taken between 1 and 7 days after stabilization of the screw (1 month/SME, 5 months/RME) SME: (0.4 mm) weekly‐ RME: (0.4 mm) per day‐	33 patients (7–10 y) Haas ‐ SME: 16 (8 m/8 f) (9 years) ‐ RME: 17 (7 m/10 f) (8.9 years) ‐ CBCT	Brunetto et al. (2013) RCT
MW/IM = 30.15%	‐ SME: IC: 1.9 ± 1.3^a^ IM: 6.3 ± 2.1^a^ MW: 1.9 ± 1.0^a^ ‐ RME: IC: 2.5 ± 1.5^a^ IM: 5.7 ± 1.6^a^ MW: 2.4 ± 0.9^a^	‐ SME 0.50 mm per week ‐ RME 0.75 mm per day ‐ In both groups, the jackscrew was activated until a 2‐mm molar transverse overcorrection was achieved without specific duration	26 patients with band palatal expander ‐ 12 SME (10.3 ± 2.5 years) ‐ 14 RME (9.7 ± 1.5 years) CT	Martina et al. (2012) RCT
—	‐ TG: IM: 4.50 ± 2.72^a^ CG: IM: 0.6 ± 1.37‐	‐ Treatment duration (14–16 m ± 8 m) ‐ Observation time (16–18 m ± 8.5 m) ‐ The appliance was activated once a month	100 patients ‐ TG: 50 (20 m/30 f) (9.5–9.6 y ± 18 m) QH ‐ CG: 50 (20 m/30 f) (9–9.7 y ± 16 m) ‐ Dental casts	Shundo et al. (2012) CCT
MW/IM = 60%	IM: 6.5 ± 2.9^a^ MW: 3.9 ± 1.8^a^	Treatment duration (6 months)‐ ‐ Activated protocol was not mentioned	73 patients (9.2–11.9 y) ‐ QH ‐ CBCT	Corbridge et al. (2011) A prospective study
—	QH: IC: 3.48 ± 2.24^a^ IM: 5.70 ± 2.31^a^ RP: IC: 1.80 ± 2.96^a^ IM: 4.46 ± 2.22^a^ CG: IC: ‐0.17 ± 0.59 IM: 0.15 ± 0.76^a^	‐ QH: Treatment duration (4.24 ± 2.05 m), it was activated once a month ‐ RP: Treatment duration (6.12 ± 3.25 m), it was activated quarter rotation a week	99 patients ‐ QH: 33 (7 m/26 f) (8 y ± 0.79) ‐ RP: 33 (15 m/18 f) (7.82 y ± 0.85) ‐ CG: 33 (19 m/14 f) (8.09 y ± 0.81) ‐ Dental casts	Godoy et al. (2011) RCT
—	‐ All three expanders IC: 4.56 ± 0.32 IM: 4.32 ± 0.4 CG: IC: 0.57 ± 0.66 IM: 0.70 ± 0.68^a^	Treatment duration: Until‐ transverse overcorrection 1 mm was achieved, but the period between T1 and T2 was 1.1 y ‐ Modified Haas: Was activated one turn (0.25 mm) every 2 days ‐ Hyrax: Was activated one turn (0.25 mm) every 2 days ‐ QH: One activation every 4–6 weeks	110 patients (7 y + 7 m) ‐ Modified Haas: 56 (18 m/38 f) ‐ Hyrax: 26 (11 m/15 f) ‐ QH: 28 (13 m/15 f) ‐ Dental casts	Wong et al. (2011) Comparative study
—	IM: ‐ Haas: 5.3 ± 1.8 ‐ Hyrax: 4.7 ± 1.3 ‐ QH: 5.1 ± 2.1	‐ Treatment duration: Until the PXB was mildly overcorrected, between T1 and T2 was 1.1 y ‐ Haas and the hyrax: 1 turn every 2 days ‐ QH: 1 molar width of activation	132 patients (8 y) ‐ Haas: 57 (8.1 ± 1.1) ‐ Hyrax: 32 (7.8 ± 1.1) ‐ QH: 43 (8.3 ± 1.0) ‐ Dental casts	Huynh et al. (2009) A retrospective study
—	QH: IC: 2.0 ± 1.18^a^ IM: 4.6 ± 1.19^a^ RP: IC: 2.7 ± 1.20^a^ IM: 3.5 ± 1.54^a^ ‐ Composite onlay: IC: 0.63 ± 0.7^a^ IM: 0.5 ± 0.46^a^ CG: IC: 0.3 ± 0.43^a^ IM: 0.4 ± 0.43^a^	QH: Was activated 10 mm before placement and then reactivated every 6 weeks ‐ RP: Was activated 0.2 mm once a week composite onlay: Was checked every 6 weeks and removed after a year ‐ In all groups no overcorrection was produced, but the period between T1 and T2 was 1 y	60 patients ‐ QH: 15 (6 m/9 f) (9.1 y ± 1.03) ‐ RP: 15 (6 m/9 f) (8.7 y ± 0.82) ‐ Composite onlay: 15(7 m/8 f) (8.3 y ± 0.70) ‐ CG: 15(7 m/8 f) (8.8 y ± 0.70) Dental casts‐	Petrén and Bondemark (2008) RCT
MW/IM = 90.68%	‐ TG: IM: 4.94 ± 1.55^a^ MW: 4.48 ± 1.96^a^ CG: IM: 1.45 ± 1.24 MW: 2.15 ± 1.54	Treatment duration‐ (10 months) ‐ The springs were activated every 3–4 weeks	43 patients ‐ TG: 23 (6 y 2 ± 17 m) RP ‐ CG: 20 (5 y 9 ± 15 m) PA cephalograms‐	Defraia et al. (2007) A retrospective case‐control study
—	IC: 4.29 ± 2.67^a^ IM: 6.83 ± 3.08^a^	‐ The appliances were active for approximately 12 months ‐ QH was activated 6 mm (3 mm per side) at 8 weeks and again about 3 months later ‐ Bonded palatal expanders were activated 1 turn per week	54 patients (25 m/29 f) (8.8 ± 1.7) ‐ QH ‐ Bonded palatal expanders Dental casts‐	Vargo et al. (2007) A retrospective study
—	NiTi: Primary dentition: ‐ IC: 0.7 ± 0.6 ‐IM: 1.0 ± 0.6 mixed dentition: ‐ IC: 1.2 ± 0.9 ‐ IM: 1.4 ± 0.9 CC: IC: 0.41 ± 0.45 IM: 0.3 ± 0.3	‐ Treatment duration (6 months) ‐ Activated protocol was not mentioned	20 patients ‐ NiTi palatal expander: ‐ Primary dentition (4 f: 5.8 y ± 0.7) ‐ Mixed dentition (6 m: 8.7 y ± 1.2/3 f: 8.7 y ± 0.6) ‐ CG: ‐ Primary dentition (1 m: 4.8 y/1 f: 4 y) ‐ Mixed dentition (3 m: 7.9 y ± 1.5/2 f: 7.4 y ± 0.3) ‐ Dental casts	Ferrario (2003) CCT
MW/IM = 16%	NiTi: IM: 6.26 ± 1.65^a^ MW: 0.99 ± 0.45^a^ RME: IM: 4.76 ± 1.55^a^‐ MW: 1.41 ± 1.09^a^	Treatment duration (4.2 months) ‐ NiTi: 153 days ‐ RME: 127 days	‐ 25 patients (9.4–11.1 y) ‐ NiTi expander: 13 patients ‐ RME: 12 patients ‐ Dental casts	Ciambotti et al. (2001) Comparative study
CG: 25% RP: 27.7% QH: 25%	CG: IC: 0.7 ± 0.5^a^ IM: 0.4 ± 0.3^a^ MW: 0.1 ± 0.2^a^ RP: IC: 1.5 ± 0.8^a^ IM: 1.8 ± 1^a^ MW: 0.5 ± 0.6^a^ QH: IC: 3.1 ± 1.9 IM: 5.6 ± 1.9^a^ MW: 1.4 ± 1.2^a^	treatment duration: ‐ CG: 0.7 y ‐ RP: 0.6 y ‐ QH: 0.6 y ‐ Activating protocol was once a month	37 patients (9.3‐9.7 y) 10 were ‐ CG: 10 patients ‐ RP: 13 patients ‐ QH: 14 patients ‐ PA cephalograms and dental casts	Erdinç et al. (1999) A retrospective study
—	SME: IC: 6.35 ± 0.45^a^ IM: 9.81 ± 0.57^a^ RME: IC: 6.36 ± 0.45^a^ IM: 9.05 ± 0.40^a^	Treatment duration: ‐ Minne: 1–5.16 m Hyrax: 0.70–1.60 m‐ ‐ Minne was compressed each week ‐ Hyrax was activated twice a day	24 patients ‐ SME: Minne expander (9.83–13.50 y) ‐ RME: Hyrax expander (10.40–13.50 y) ‐ Dental casts	Akkaya (1998) Comparative study
MW/IM = 35.84%	‐ SME: IC: 4.9 ± 3.9^a^ IM: 5.3 ± 2.6^a^ MW: 1.9 ± 1.3^a^ ‐ SRME: IM: 4 ± 2.2^a^ MW: 1.5 ± 2^a^ ‐ RME: IM: 6.2 ± 2.6^a^ MW: 2.7 ± 1.2^a^	‐ SME: 56 d = 1.9 m The appliance was activated weekly ‐ SRME: 5.5 m ‐ RME: 19.2 d	30 patients (6.6–8.9 y) ‐ SME: 10 QH ‐ SRME: 10 RP ‐ RME: 10 Hyrax ‐ PA cephalograms	Sandikçiolu and Hazar (1997) Comparative study
MW/IM = 24%	QH: IM: 5.4 ± 2.0^a^ MW: 1.3 ± 0.9^a^ RME: IM: 6 ± 2.6^a^ MW: 2.6 ± 1.4^a^	Treatment duration ‐ QH (149 d = 5 m) ‐ RME (23 d)	60 patients (11.8–11.11 y) ‐ QH: 30 patients ‐ RME: 30 patients Dental casts	Ladner and Muhl (1995) A retrospective study
MW/IM: ‐ QH = 14.3% ‐ RP = 19.6%	QH: IC: 5.17 ± 1.86^a^ IM: 5.61 ± 1.78 RP: IC: 3.50 ± 1.25 IM: 4.65 ± 1.52	Treatment duration: ‐ QH: 42–147 days (mean: 101.2 d) ‐ RP: 72–228 days (mean 115.4 d) activated protocol: ‐ QH: Was expanded every third week and no extra activation was performed. ‐ RP: Was turned twice a week	34 patients ‐ QH: 17 (6.7–11.3 years) ‐ RP: 17 (6.4–10.9 years) ‐ PA cephalograms and dental casts	Boysen et al. (1992) CCT
—	1st: IC: 6.4 ± 1.1 IM: 7.9 ± 1.5 2nd: IC: 5.3 ± 1.9 IM: 5.3 ± 1.1	Treatment duration: In both groups 7–15 weeks Activated protocol: In both groups: The spring was reactivated every week	10 patients (6 m/4 f) ‐ Minne expander: 5 (8 y 7 m to 10 y 9 m) ‐ Minne expander soldered to bands: 5 (8 y 8 m to 12 y) ‐ PA cephalograms and dental casts	Mossaz‐Joëlson and Mossaz (1989) Comparative study
—	Deciduous‐dentition: IC: 3.9 ± 0.8^a^ IM: 5.7 ± 0.5^a^ mixed‐dentition: IC: 4.4 ± 5 0.7^a^ IM: 4.8 ± 1.3^a^	Treatment duration: ‐ Deciduous‐dentition: (14–42 d) with a mean of (28.8 ± 4.9 days) ‐ Mixed‐dentition: (14–42 d)with a mean of (31.8 ± 5.9 d) Activated protocol: Initial expansion was equivalent to a distance one‐half the buccolingual width of the molars	10 patients (4 y 5 m to 9 y 3 m) ‐ 5 patients: Deciduous‐dentition: 5 (5 y 3 m) ‐ 5 patients: Mixed‐dentition 5 (8 y 2 m) ‐ QH ‐ Dental casts and intraoral photographs	Bell and LeCompte (1981) Comparative study
MW/IM = 15.64%	IC: 2.74 ± 3.23^a^ IM: 5.88 ± 3.64^a^ MW: 0.92 ± 1.59^a^	‐ Treatment duration (9 months) ‐ Activated protocol was not mentioned	20 patients (10 y ± 3 m) QH ‐ PA cephalograms and dental casts	Frank and Engel (1982) A prospective study

a Statistically significant difference; ‐, the ratio of MW/IM cannot be calculated.

### 3.3 Risk of Bias in Individual Studies and Across Studies

Figures 2 and 3 show the risk of bias of the individual studies. Five RCTs were assessed as having a low risk of bias (Godoy et al. 2011; Petrén and Bondemark 2008; Martina et al. 2012; Brunetto et al. 2013; Serafin et al. 2022). Two RCTs were judged to have some bias (Nagrik and Bhad 2014; Paoloni et al. 2022). In addition, no other risk factor was evaluated as a high risk of bias. Allocation concealment was unclear in 14.2% of the studies. There was bias in the measurement of outcomes in 28.5% of the included RCTs. According to the ROBINS‐I tool, which was applied for 21 studies (Frank and Engel 1982; Bell and LeCompte 1981; Boysen et al. 1992; Ladner and Muhl 1995; Sandikçiolu and Hazar 1997; Erdinç et al. 1999; Vargo et al. 2007; Huynh et al. 2009; Wong et al. 2011; Corbridge et al. 2011; Shoaib et al. 2017; Defraia et al. 2007; Van de Velde et al. 2021; Zhang et al. 2023; Mossaz‐Joëlson and Mossaz 1989; Akkaya 1998; Ciambotti et al. 2001; Ferrario 2003; Brunetto et al. 2013; Bukhari et al. 2018; Cossellu et al. 2020), 8 studies were at low (Ladner and Muhl 1995; Erdinç et al. 1999; Huynh et al. 2009; Shundo et al. 2012; Van de Velde et al. 2021; Zhang et al. 2023; Akkaya 1998; Bukhari et al. 2018), 4 studies were at moderate (Boysen et al. 1992; Shoaib et al. 2017; Defraia et al. 2007; Cossellu et al. 2020), 3 studies were at serious (Frank and Engel 1982; Corbridge et al. 2011; Mossaz‐Joëlson and Mossaz 1989), and 6 studies were at critical risk of bias (Bell and LeCompte 1981; Sandikçiolu and Hazar 1997; Vargo et al. 2007; Wong et al. 2011; Ciambotti et al. 2001; Ferrario 2003). Serious studies were re‐assessed using the NIH quality assessment tool for before‐and‐after studies (Pre, Post), and two of them were of good quality (Frank and Engel 1982; Corbridge et al. 2011). Critical studies were excluded from the quantitative analysis.

**Figure 2:**
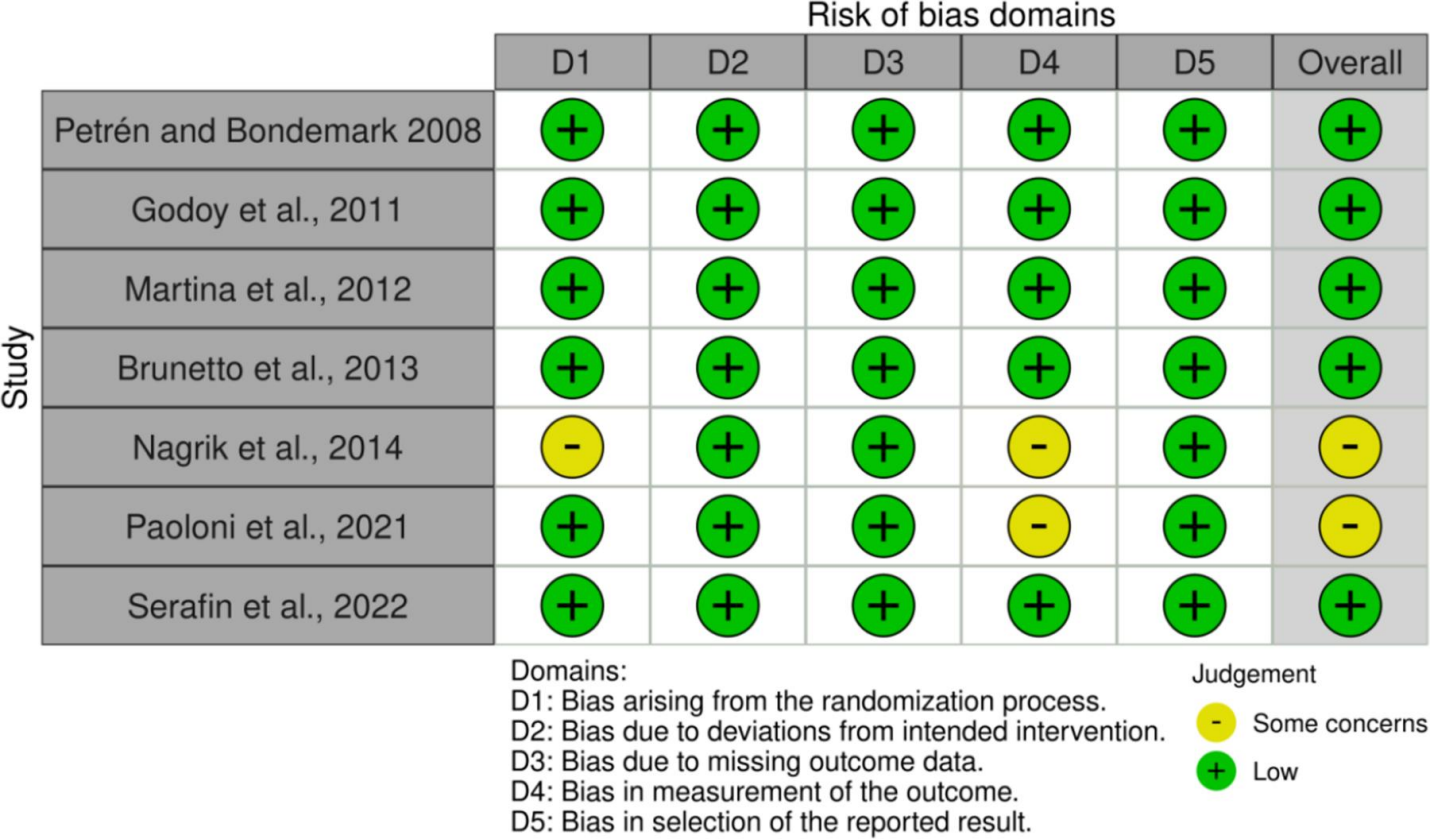
Risk of bias summary for the randomized controlled studies included.

**Figure 3:**
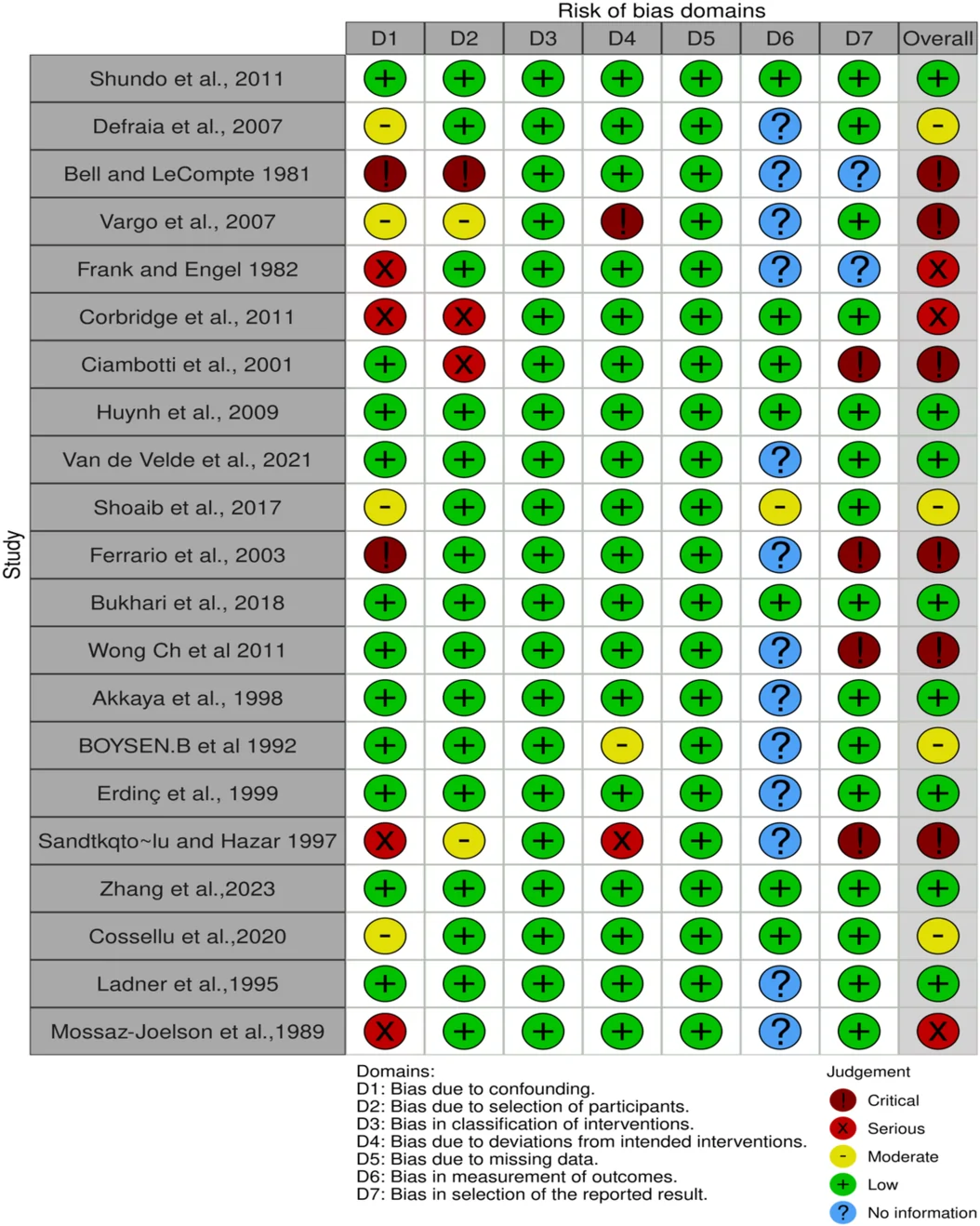
Risk of bias summary for the nonrandomized studies included.

However, most studies included QH and removable plate (RP), while few studies evaluated other types of expanders. Therefore, the focus of our review was on the largest number of studies, which contained QH and RP (Supporting Material 1).

### 3.4 Effects of Interventions: Results of the Individual Studies (Quantitative Analysis) and Meta‐Analysis

According to the available data in this systematic review, three themes of comparisons could be made:

‐ Pre versus post treatment (T0–T1) comparisons within expansion groups.

‐ Comparisons of the treatment effects between groups.

‐ Comparison between the RP and the untreated groups (CG).

Also, publication bias was assessed using funnel plots (Supporting Material 2).

### 3.5 First: The QH Appliance

#### 3.5.1 Qualitative Findings

IM width ranged from 4.32 mm (Wong et al. 2011) to 10.1 mm (Shoaib et al. 2017). Nine studies (two RCTs, seven non‐RCTs) were pooled into the meta‐analysis (Godoy et al. 2011; Erdinç et al. 1999; Vargo et al. 2007; Petrén and Bondemark 2008; Huynh et al. 2009; Wong et al. 2011; Corbridge et al. 2011; Shundo et al. 2012; Shoaib et al. 2017).

#### 3.5.2 Meta‐Analysis Findings

The primary analysis revealed a significant expansion of IM width of WMD = 5.74 mm (*Z* = 28.46; *p* = 0.0001; *I* ^2^ = 75%) (Figure 4A). After the stratified analysis was performed, both non‐RCTs and RCTs showed a significant increase in IM width with a difference in heterogeneity, whereas it was substantial in non‐RCTs WMD = 5.81 mm (*Z* = 26.45; *p* = 0.0001; *I* ^2^ = 80%) but not significant in RCTs WMD = 5.39 mm (*Z* = 10.52; *p* = 0.33; *I* ^2^ = 0%) (Figure 4B). Stratified analysis demonstrated that the high heterogeneity observed in the primary analysis was entirely driven by a specific subset of studies. After stratifying the analysis and removing these heterogeneous studies, the *I* ^2^ value decreased from high levels to 0%, indicating complete elimination of statistical heterogeneity. Importantly, the pooled effect size remained consistently high both before‐and‐after stratification, demonstrating that the overall magnitude of the treatment effect was robust and not materially influenced by the exclusion of the heterogeneous studies. These findings suggest that while the initial heterogeneity was study‑specific, the core effect estimate is stable and reliable. The IC width ranged from 2 mm (Petrén and Bondemark 2008) to 5.17 mm (Boysen et al. 1992); five studies (two RCTs, three non‐RCTs) were included in the meta‐analysis (Godoy et al. 2011; Erdinç et al. 1999; Vargo et al. 2007; Petrén and Bondemark 2008; Wong et al. 2011). It increased significantly with significant heterogeneity; WMD = 3.78 mm (*Z* = 14.41; *p* = 0.006; *I* ^2^ = 73%) (Figure 5A). Both the non‐RCTs and the RCTs showed a significant increase in IC width after sensitivity analysis was performed, but the heterogeneity was not significant in the non‐RCTs WMD = 4.42 mm (*Z* = 13.26; *p* = 0.42; *I* ^2^ = 0%) or RCTs WMD = 2.74 mm (*Z* = 6.45; *p* = 0.08; *I* ^2^ = 68%) (Figure 5B). The effect size remained high across all analyses, while heterogeneity dropped from a high level to zero after stratifying the studies, indicating that the overall effect was strong and stable despite the initial variability. The means of the MW achieved through QH appliances were between 0.9 and 3.9 mm (Frank and Engel 1982; Corbridge et al. 2011). Three articles (non‐RCTs) were pooled to estimate the MW and the differences caused by expansion (Erdinç et al. 1999; Corbridge et al. 2011; Shoaib et al. 2017). Figure 6 provides graphical representations of the MW, which was significantly expanded, with a mean of WMD = 3.42 mm (*Z* = 9.74; *p* = 0.02; *I* ^2^ = 74%).

**Figure 4:**
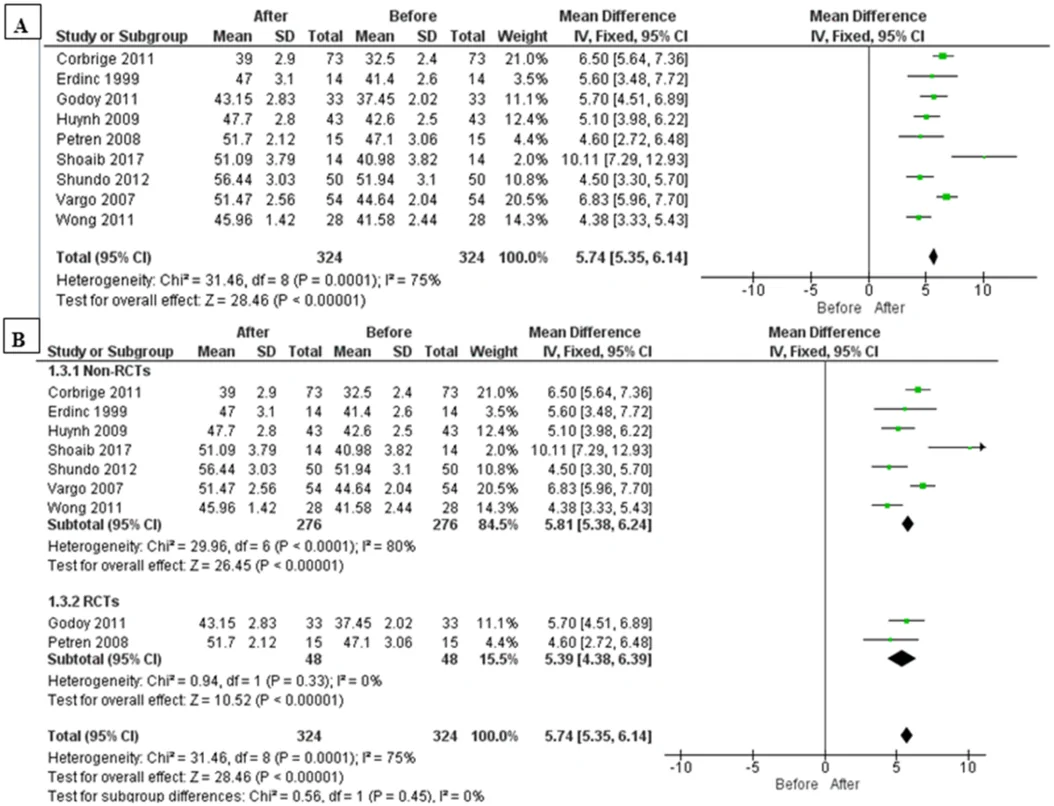
Forest plots of comparison: comparison of QH treatment, outcome: maxillary inter‐molar width (IM) (mm). (A) Without stratified analysis. (B) With stratified analysis.

**Figure 5:**
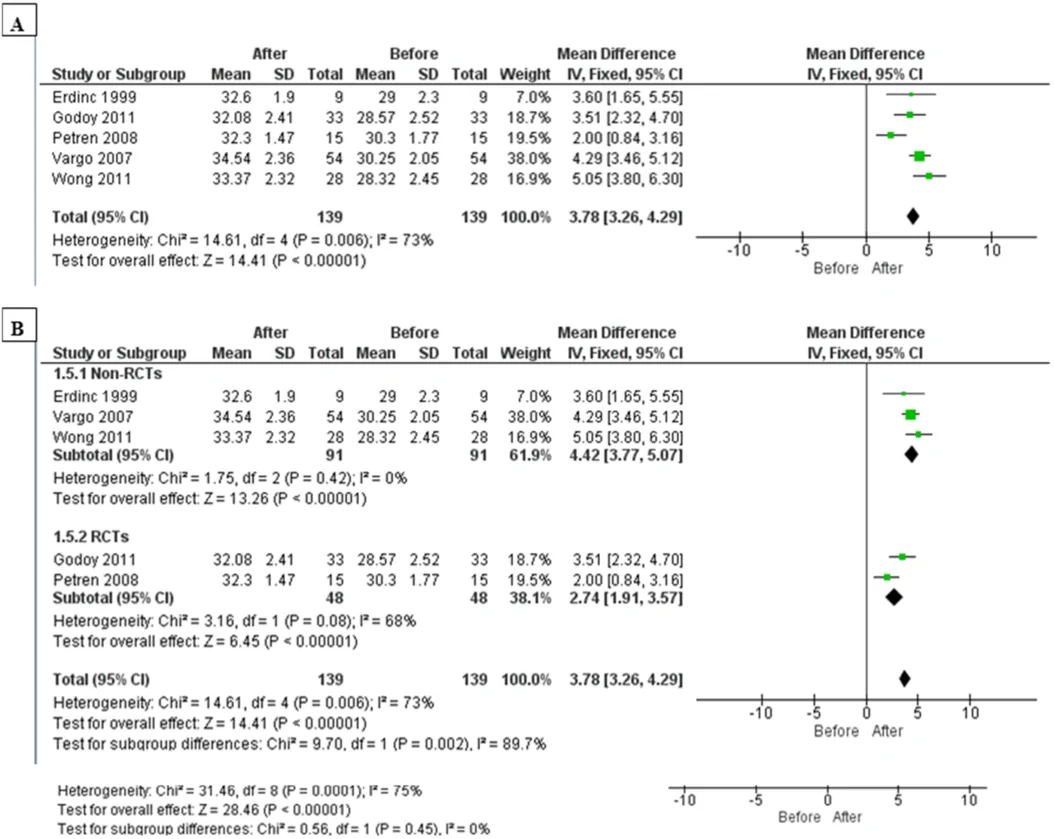
Forest plots of comparison: comparison of QH treatment, outcome: maxillary inter‐canine width (IC) (mm). (A) Without stratified analysis. (B) With stratified analysis.

**Figure 6:**
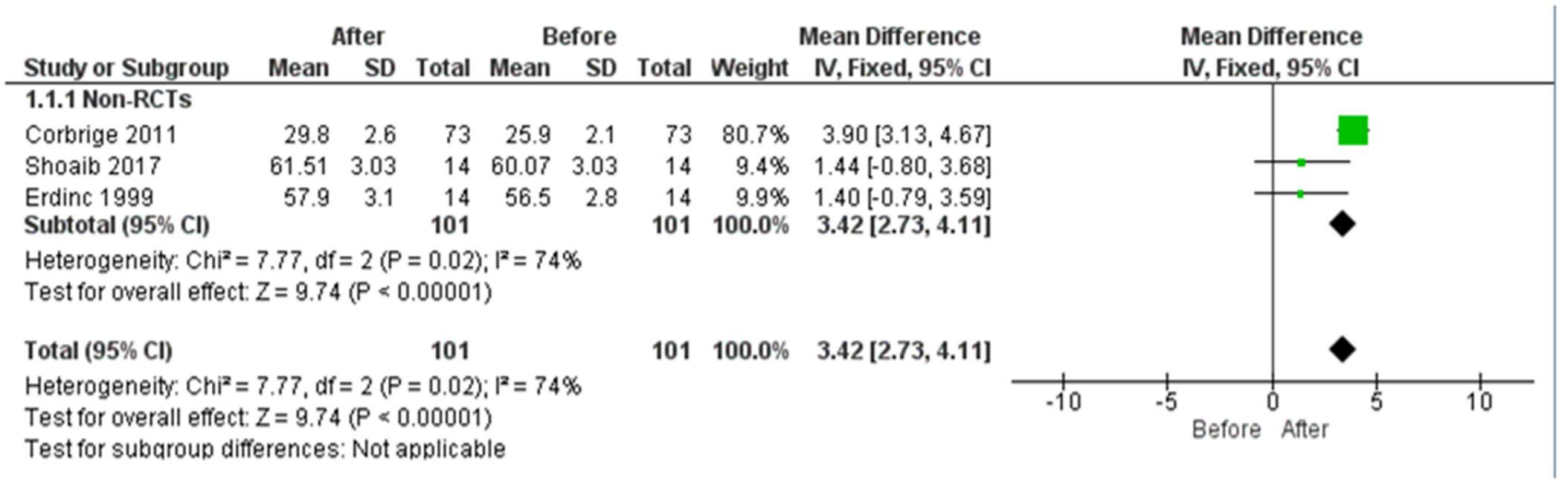
Forest plots of comparison: comparison of QH treatment, outcome: maxillary width (MW) (mm).

### 3.6 Second: RP Appliance

#### 3.6.1 Qualitative Findings

The IM width was 3.5 mm in Petrén and Bondemark (2008) and Van de Velde et al. (2021), and 4.94 mm in Defraia et al. (2007). Five studies (two RCTs, three non‐RCTs) were pooled into the meta‐analysis (Godoy et al. 2011; Erdinç et al. 1999; Petrén and Bondemark 2008; Van de Velde et al. 2021; Zhang et al. 2023).

#### 3.6.2 Meta‐Analysis Findings

The primary analysis revealed a significant increase in the U6–U6 width, WMD = 3.61 mm (*Z* = 18.07; *p* = 0.78; *I* ^2^ = 0%) (Figure 7A). After the stratified analysis was performed, the overall effect also revealed a significant increase (*p* < 0.00001) (Figure 7B). Considering the *I* ^2^ values before and after stratifying the studies, the heterogeneity ratio remained zero = 0, indicating that the results are consistent and highly reliable. Additionally, the IC widths were 1.86 mm (15) and 3.8 mm (Van de Velde et al. 2021). Five studies (two RCTs, three CCTs) were included in the meta‐analysis (Godoy et al. 2011; Erdinç et al. 1999; Petrén and Bondemark 2008; Van de Velde et al. 2021; Zhang et al. 2023). It increased significantly with significant heterogeneity; WMD = 3.51 mm (*Z* = 18.38; *p* = 0.07; *I* ^2^ = 53%) (Figure 8A). The *I* ^2^ = 53% before stratifying the studies, but decreased to zero after separating both RCT and non‐RCT studies, indicating a complete resolution of variability and supporting the reliability of the results. However, after the stratified analysis was performed, both non‐RCTs and RCTs showed a significant increase in the IC width with low heterogeneity (Figure 8B). Seven studies involved removable appliances; the amount of maxillary expansion ranged from 1.1 mm (Erdinç et al. 1999) to 4.48 mm (Defraia et al. 2007). Two articles (non‐RCTs) were pooled to estimate the MW and the differences caused by expansion (Erdinç et al. 1999; Zhang et al. 2023). Figure 9 provides graphical representations of the MW width, which was significantly expanded, with a mean of 1.71 mm WMD = 1.71 mm (*Z* = 3.76; *p* = 0.47; *I* ^2^ = 0%).

**Figure 7:**
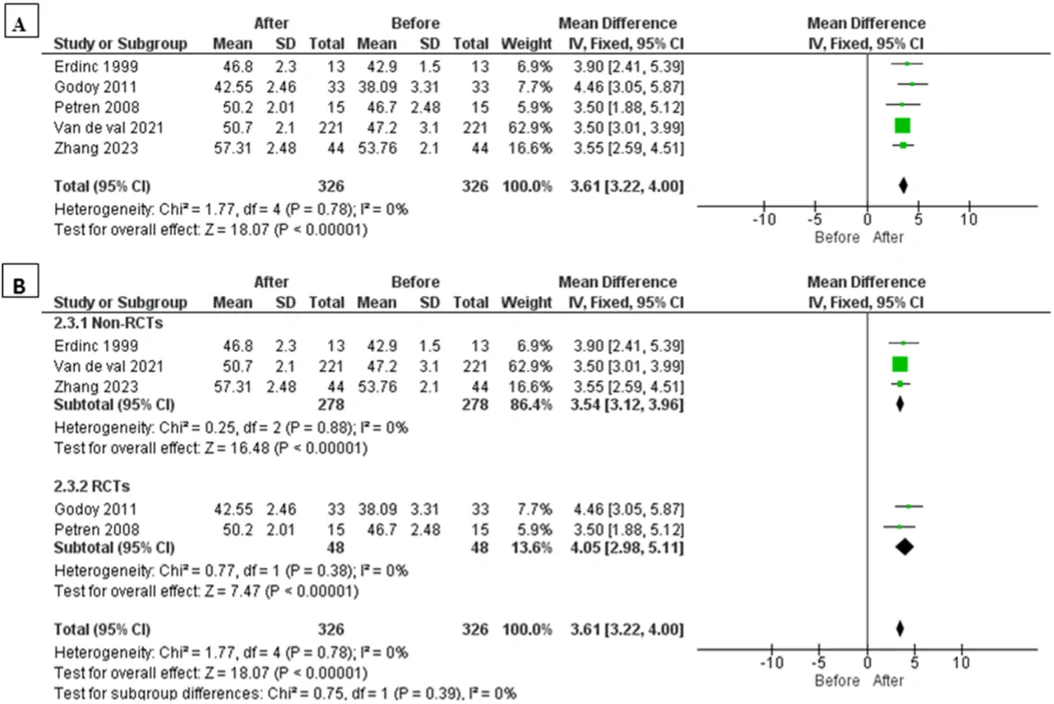
Forest plots of comparison: comparison of RP treatment, outcome: maxillary inter‐molar width (IM) (mm). (A) Without stratified analysis. (B) With stratified analysis.

**Figure 8:**
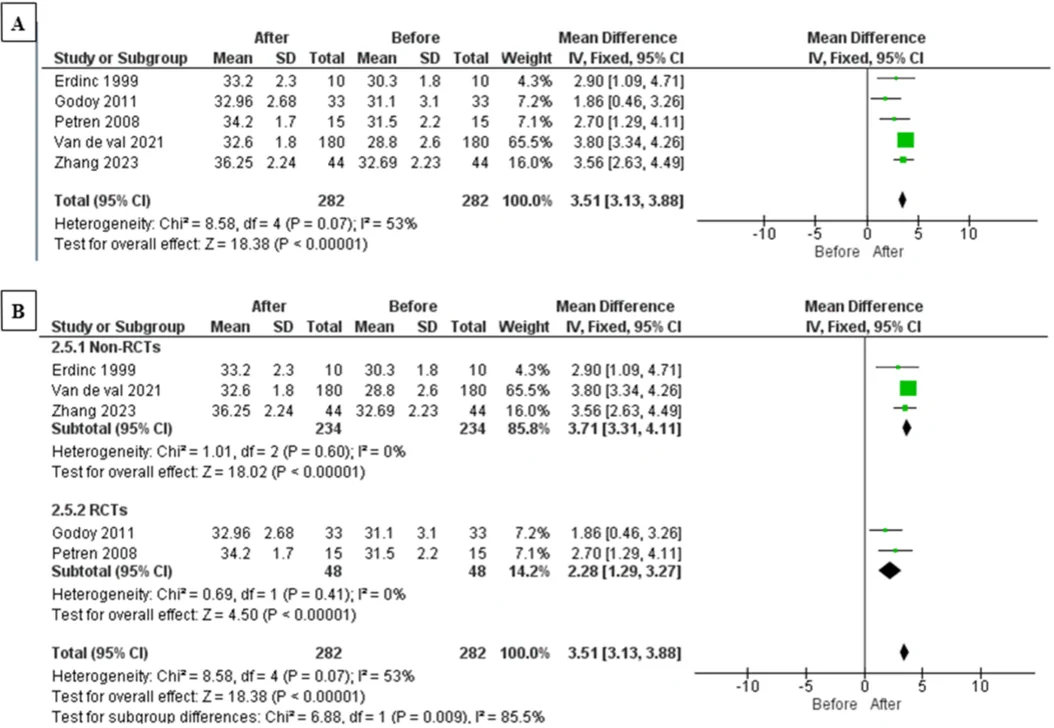
Forest plots of comparison: comparison of RP treatment, outcome: maxillary inter‐canine width (IC) (mm). (A) Without stratified analysis. (B) With stratified analysis.

**Figure 9:**
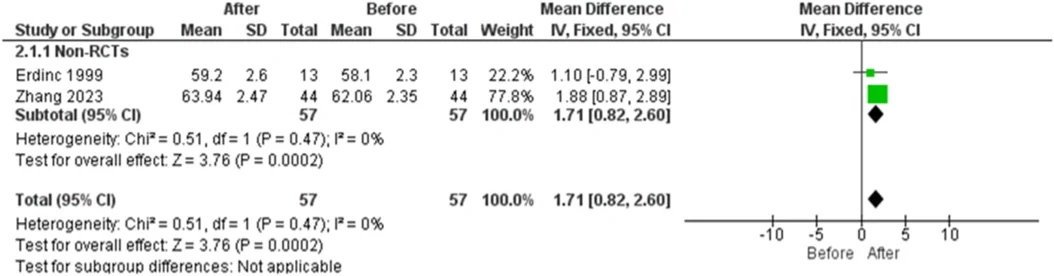
Forest plots of comparison: comparison of RP treatment, outcome: maxillary width (MW) (mm).

### 3.7 Third: The QH and the CG

#### 3.7.1 Qualitative Findings

In this systematic review, four studies (two RCTs, two non‐RTCs) used QH and compared the results with those of the control group. One study reported a significant increase in MW (Erdinç et al. 1999). Three studies (two RCTs, one non‐RCT) reported a substantial increase in the IC width of the maxillary arch (mean value: 3.1, 2, 3.46 mm) compared with that of the control group (mean value: 0.7, 0.3, 0.17 mm) (Godoy et al. 2011; Erdinç et al. 1999; Petrén and Bondemark 2008).

#### 3.7.2 Meta‐Analysis Findings

The primary analysis revealed a significant difference in IC width between the QH group and the control group WMD = 2.46 mm (*Z* = 10.41; *p* = 0.0008; *I* ^2^ = 86%) (Figure 10A), and after the applied stratified analysis, WMD = 2.47 mm (*Z* = 9.76; *p* = 0.00002; *I* ^2^ = 93%) (Figure 10B). All of these methods significantly increased the IM width (4.5, 5.6, 4.6, and 5.7 mm) compared with that of the control group (0.6, 0.4, 0.4, and 0.16 mm) (Godoy et al. 2011; Erdinç et al. 1999; Petrén and Bondemark 2008; Shundo et al. 2012). The primary analysis revealed a significant difference between the QH group and the control group WMD = 4.60 mm (*Z* = 22.58; *p* = 0.01; *I* ^2^ = 71%) (Figure 11A). After the stratified analysis was performed, WMD = 4.43 mm (*Z* = 13.40; *p* = 0.05; *I* ^2^ = 73%), and there was no significant difference between the results of the RCTs and non‐RCTs (Chi2 = 0.42, df = 1 [*p* = 0.52], *I* ^2^ = 0%; Figure 11B). In both of IM and IC width, the *I* ^2^ remained high before and after stratifying the studies, while the effect size consistently remained high across all analyses. This indicates substantial heterogeneity that did not materially affect the magnitude of the pooled effect and may that high heterogeneity indicates differences in the follow‐up periods.

**Figure 10:**
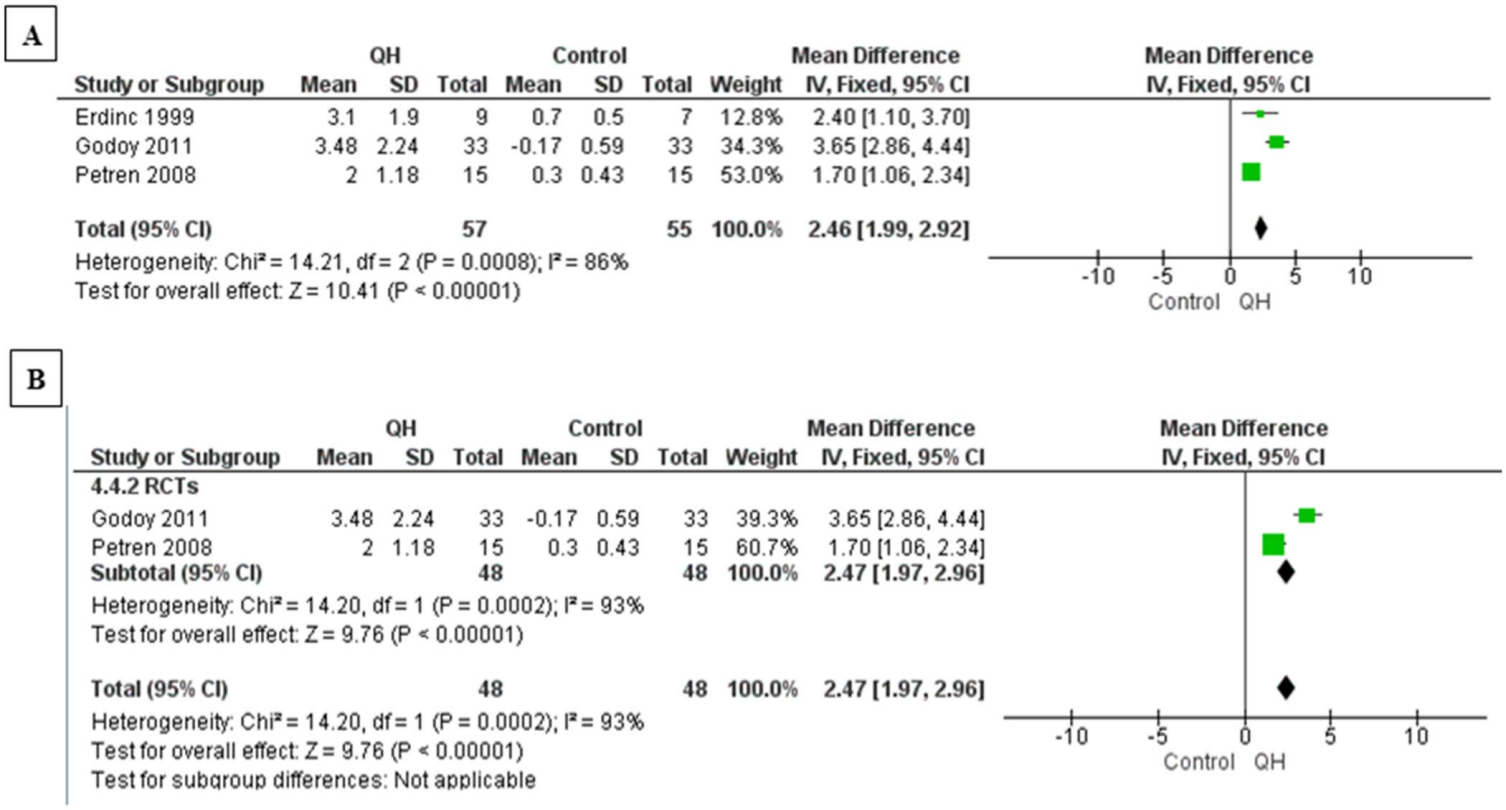
Forest plots of comparison: comparison between QH and control group, outcome: maxillary inter‐canine width (IC) (mm). (A) Without stratified analysis. (B) With stratified analysis.

**Figure 11:**
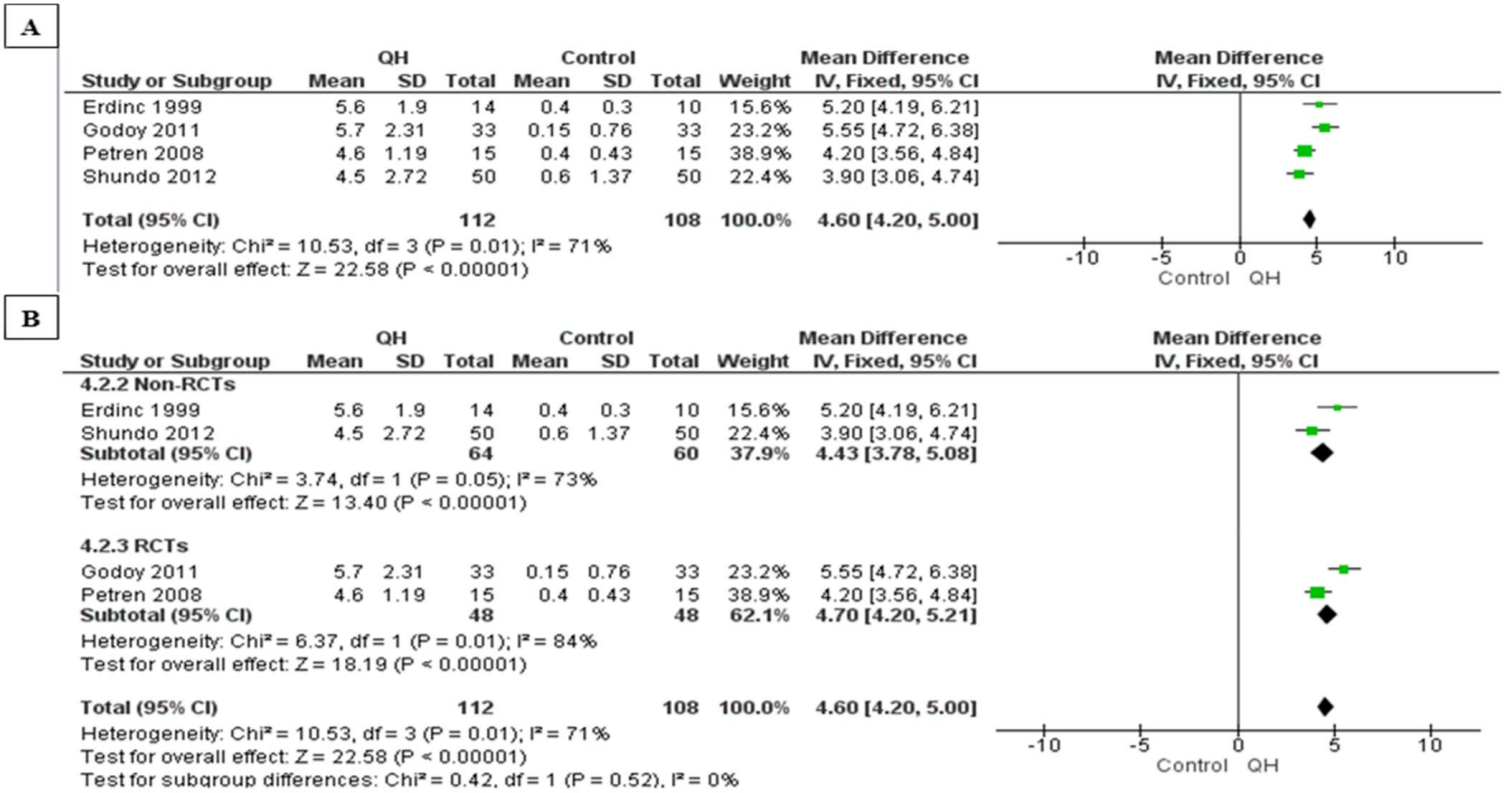
Forest plots of comparison: comparison between QH and control group, outcome: maxillary inter‐molar width (IM) (mm). (A) Without stratified analysis. (B) With stratified analysis.

### 3.8 Fourth: The RP and the CG

#### 3.8.1 Qualitative Findings

Additionally, four studies (two RCTs, two non‐RCTs) used RP and compared their results with those of the control group. Two studies measured MW and found a significant increase in it (Erdinç et al. 1999; Defraia et al. 2007). All of them significantly increased the IM width (1.8, 4.94, 3.5, and 4.46 mm) compared with those of the control group (0.4, 1.45, 0.4, and 0.15 mm) (Godoy et al. 2011; Erdinç et al. 1999; Petrén and Bondemark 2008; Defraia et al. 2007).

#### 3.8.2 Meta‐Analysis Findings

The primary analysis revealed a significant difference in the IM width between the RP group and the control group WMD = 2.57 mm (*Z* = 12.44; *p* = 0.00001; *I* ^2^ = 94%) (Figure 12A). After the stratified analysis was performed, there was also a significant difference between the groups (*p* = 0.00001) with effect size consistently remained high across all analyses WMD = 3.71 mm (*Z* = 12.78; *p* = 0.04; *I* ^2^ = 77%) (Figure 12B). Heterogeneity remained high both before and after stratifying the studies by design. Although separating studies (RCT, non‐RCT) helps reduce part of the variability, substantial heterogeneity persisted because the included studies differed markedly in treatment duration and the timing of outcome assessment. These two factors varied widely across studies, which explains why the *I* ^2^ values remained elevated even after sensitivity analysis. Three studies (two RCTs, one non‐RCT) reported a significant increase in the maxillary IC width (mean value: 1.5, 2.7, and 1.8 mm) compared with the control group (0.7, 0.3, and 0.17 mm) (Godoy et al. 2011; Erdinç et al. 1999; Petrén and Bondemark 2008). The primary analysis revealed a significant difference between the RP group and the control group, with significant heterogeneity WMD = 1.66 mm (*Z* = 7.80; *p* = 0.002; *I* ^2^ = 84%) (Figure 13A). However, after the stratified analysis was performed, the overall effect revealed a significant difference between the two groups (*p* = 0.00001) without any heterogeneity WMD = 2.28 mm (*Z* = 8.17; *p* = 0.49; *I* ^2^ = 0%) (Figure 13B). Therefore, the non‐RCTs were not included to obtain more accurate results.

**Figure 12:**
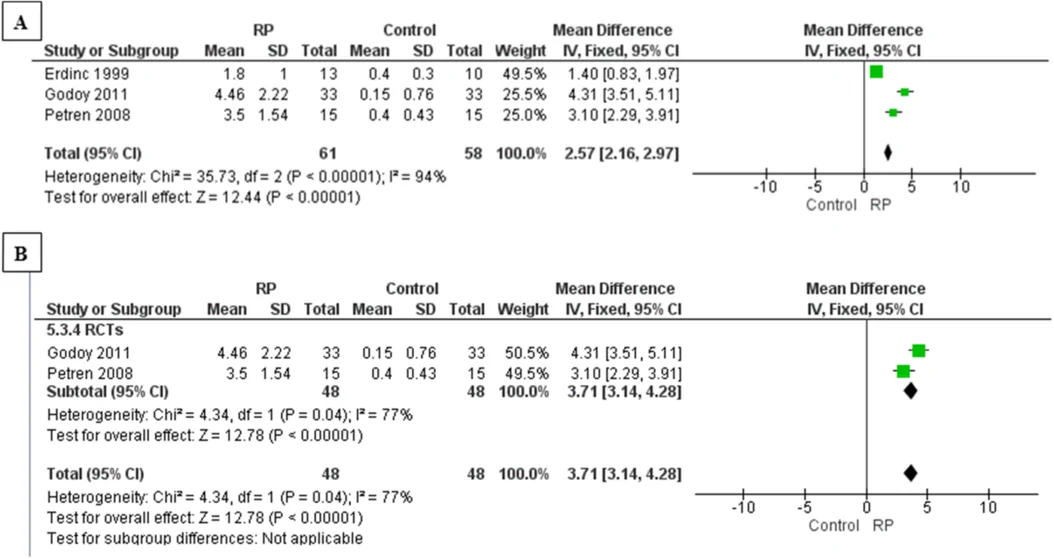
Forest plots of comparison: comparison between RP and control group, outcome: maxillary inter‐molar width (IM) (mm). (A) Without stratified analysis. (B) With stratified analysis.

**Figure 13:**
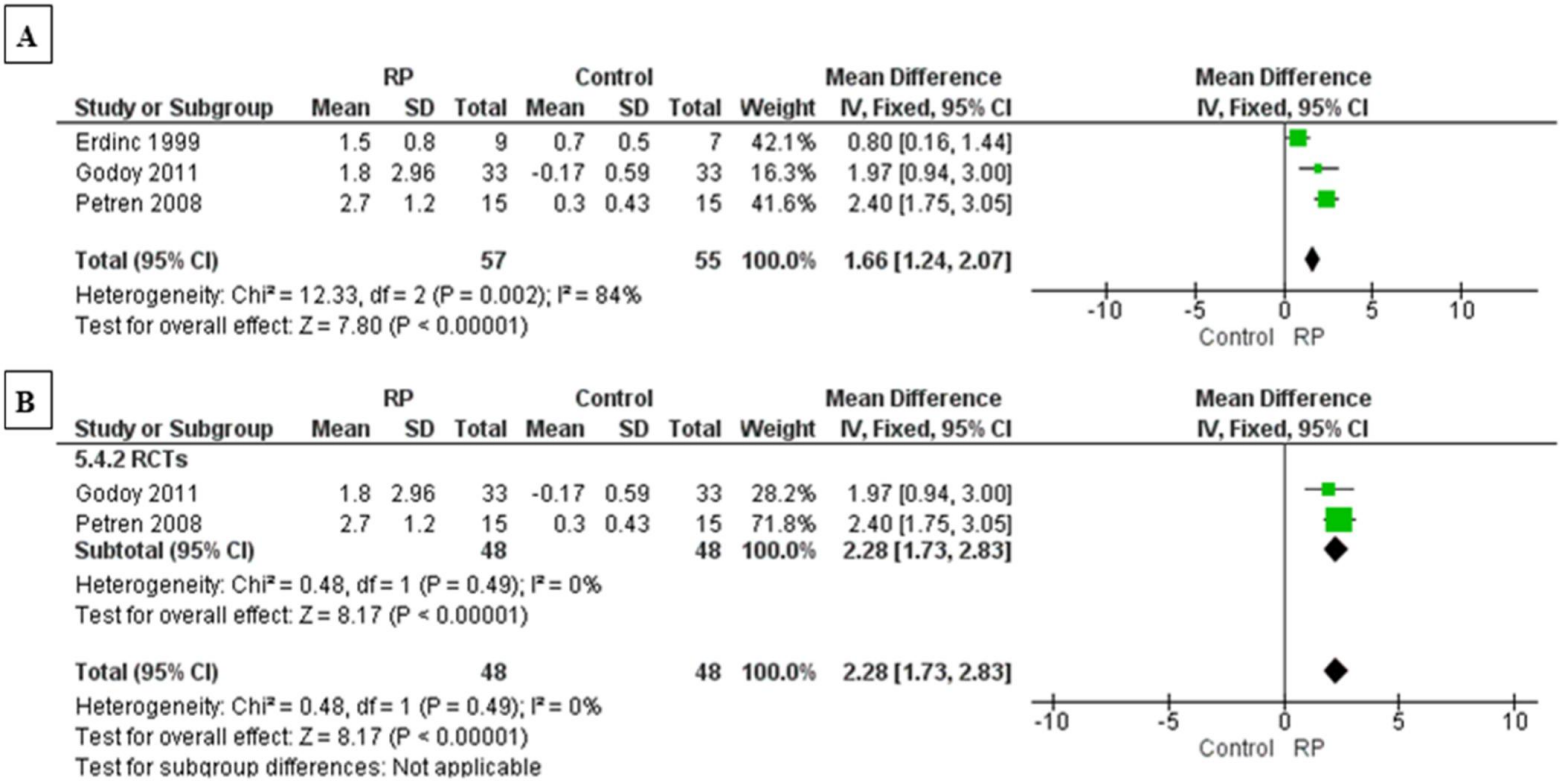
Forest plots of comparison: comparison between RP and control group, outcome: maxillary inter‐canine width (IC) (mm). (A) Without stratified analysis. (B) With stratified analysis.

### 3.9 Fifth: The QH Versus the Removable Expansion Plate

Four articles (two RCTs, two non‐RCTs) studied maxillary dental dimensions and the differences resulting from expansion (Godoy et al. 2011; Boysen et al. 1992; Erdinç et al. 1999; Petrén and Bondemark 2008). A greater increase in the maxillary IM width was found in the QH group (IM = 5.7 mm) (Zuccati et al. 2013) than in the removable expansion plate group (4.65 mm) (Boysen et al. 1992). Also, the maxillary IC width was greater in the QH group (5.17 mm) than in the RP group (3.5 mm) (Sandikçiolu and Hazar 1997), but this difference was not significant. Only one study reported an increase in the MW, which was 1.4 mm in the QH and 1.1 mm in the RP (Erdinç et al. 1999).

#### 3.9.1 Meta‐Analysis Findings

The primary analysis revealed a difference in the IM width between the QH and RP group WMD = 1.71 mm (*Z* = 6.23; *p* = 0.0007; *I* ^2^ = 82%) (Figure 14A). When stratified analyses were applied a greater increase in the maxillary IM width was found in the QH group than in the removable expansion plate group, and the difference was statistically significant between the results of the RCTs WMD = 1.16 mm (*Z* = 3.11; *p* = 0.85; *I* ^2^ = 0%) and non‐RCTs WMD = 2.35 mm (*Z* = 5.81; *p* = 0.0005; *I* ^2^ = 92%) (Figure 14B). Whereas randomized studies revealed substantive differences. In terms of the maxillary IC width and the changes that occurred due to maxillary expansion by either QH or RP, the pooled estimate revealed a statistically significant difference in the IC width between QH and RP WMD = 0.71 mm (*Z* = 2.59; *p* = 0.0005; *I* ^2^ = 83%) (Figure 15A). However, after the stratified analysis was performed, there was a significant difference between the results of the RCTs WMD = 0.04 mm (*Z* = 0.11; *p* = 0.002; *I* ^2^ = 89%) and non‐RCTs WMD = 1.64 mm (*Z* = 3.86; *p* = 0.94; *I* ^2^ = 0%) (Figure 15B). The heterogeneity coefficient (*I* ^2^) also differed after sensitivity analyses were performed, which may affect the evaluation of inconsistency during the assessment process of the level of evidence. Therefore, it was decided not to include non‐RCTs with RCTs for more reliable results of IM width.

**Figure 14:**
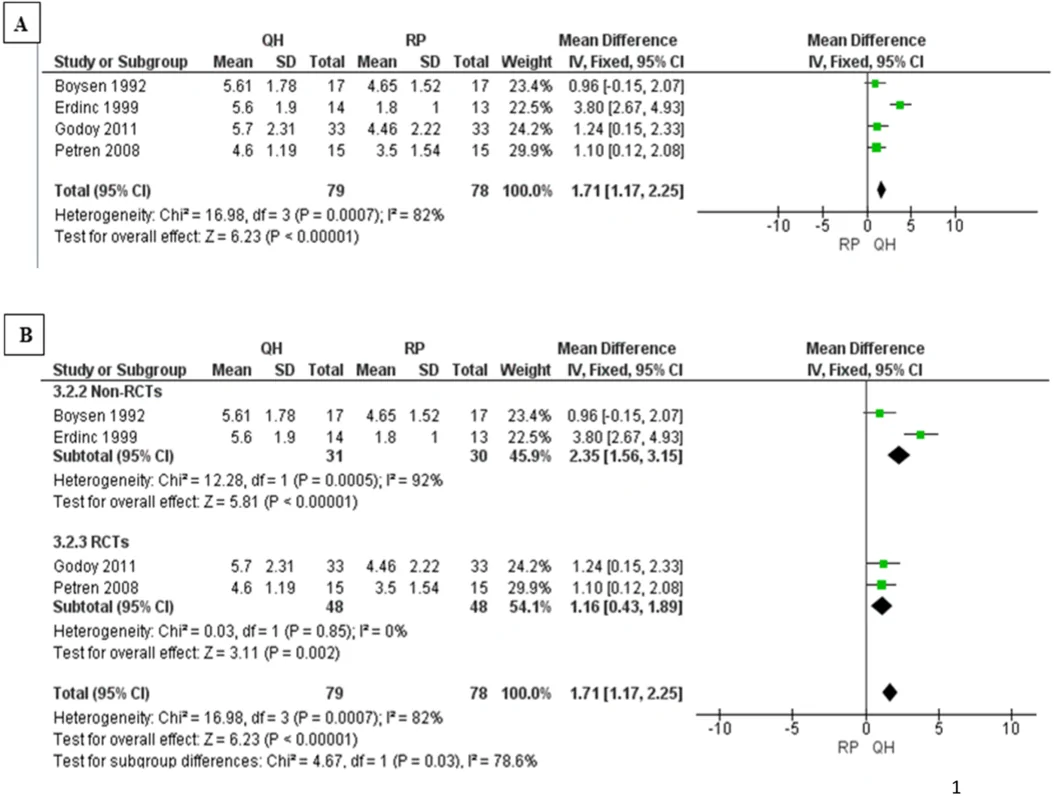
Forest plots of comparison: comparison between QH and RP, outcome: maxillary inter‐molar width (IM) (mm). (A) Without stratified analysis. (B) With stratified analysis.

**Figure 15:**
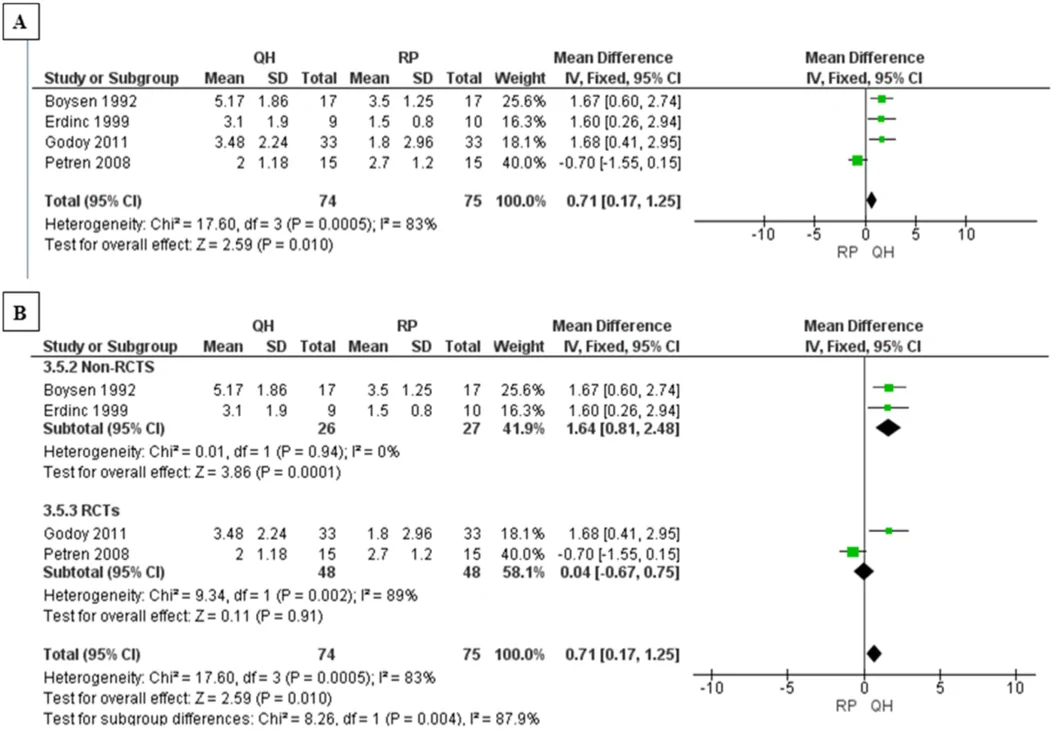
Forest plots of comparison: comparison between QH and RP, outcome: maxillary inter‐canine width (IC) (mm). (A) Without stratified analysis. (B) With stratified analysis.

### 3.10 Treatment Duration

The duration of treatment with the QH ranged from 1.06 to 13 months (Bell and LeCompte 1981; Shundo et al. 2012), whereas the duration of expansion with the removable expansion plate ranged from 3.84 to 12 months (Petrén and Bondemark 2008; Mossaz‐Joëlson and Mossaz 1989). There was only one RCT that compared the treatment duration between the QH and the RP and reported that the QH corrected the crossbite faster than the RP did, with a significant difference (Godoy et al. 2011).

### 3.11 The Strength of Evidence in the Collected Data

According to the GRADE analysis and for the first set of treatments by the QH, the level of evidence was high for RCTs of IM and moderate for RCTs of IC width, low for non‐RCTs of IC width, and very low for non‐RCTs of IM and MW (Table 3). Additionally, it was moderate for RCTs of IM and IC width and low for non‐RTCs of IM width compared with the untreated control group (Table 4). For treatment with RP, the level of evidence was high for RCTs of IM and IC width and moderate for non‐RTCs of IM, canine, and MW (Table 5). Additionally, it was moderate for RCTs of IM width and high for RCTs of IC width compared with the untreated control group. (Table 6). The comparisons between the QH and the RP revealed that the level of evidence was high for RCTs of the IM width, very low for non‐RCTs in the IM width, canine width, and low for RCTs of the IC width (Table 7).

**Table 3:** Summary of finding according to GRADE.

Quad–helix compared before and after for treatment of maxillary deficiency							
Patient or population: Patients in mixed dentition had maxillary deficiency Intervention: Quad–helix Comparison: Before and after treatment							
				Anticipated absolute effects* (95% CI)			
Comments	Certainty of the evidence (GRADE)	No. of participants (studies)	Relative effect (95% CI)	Risk after treatment	Risk before treatment		Outcomes
The treatment by quad–helix increased the maxillary inter‐molar width by 5.39 mm with *I* ^2^ = 0 between the included studies	⊕⊕⊕⊕ High	96 (2 RCTs)	—	MD 5.39 higher (4.38 lower to 6.39 higher)			Maxillary inter‐molar width—RCTs
The treatment by quad–helix increased the maxillary inter‐molar width by 5.81 mm but with large heterogeneity between the included studies	⊕◯◯◯ Very low^a^^,^ b ^,^ c ^,^ d ^,^ e	552 (7 non‐ RCTs)	—	MD 5.81 higher (5.38 lower to 6.24 higher)			Maxillary inter‐molar width—Non‐RCTs
The treatment by quad–helix increased the maxillary inter‐canine width by 2.74 mm without significant heterogeneity between the included studies	⊕⊕⊕◯ Moderate^d^	96 (2 RCTs)	—	MD 2.74 higher (1.91 lower to 3.57 higher)			Maxillary inter‐canine width—RCTs
Quad–helix increased the maxillary inter‐canine width by 4.42 mm with *I* ^2^ = 0 between the included studies	⊕⊕◯◯ Low^a^^,^ e	182 (3 non‐RCTs)	—	MD 4.42 higher (3.77 lower to 5.07 higher)			Maxillary inter‐canine width— Non‐RCTs
The evidence suggests that quad–helix increased the maxillary width by 3.42 mm	⊕◯◯◯ Very low^b^^,^ c ^,^ d ^,^ e	202 (3 non ‐RCTs)	—	MD 3.42 higher (2.73 lower to 4.11 higher)			Maxillary width—Non‐RCTs

^a^ Critical risk of bias for deviation from intended intervention in one, and for reporting bias in another one.

^b^ Serious risk of bias for confounding and selection bias in one included trial.

^c^ Moderate risk of bias for confounding bias and measurement of outcome in one included trial.

^d^ There was a high heterogeneity between the included studies.

^e^ No information on risk of bias for measurement of outcomes in one included study.

**Table 4:** Summary of findings according to GRADE.

Quad–helix compared with untreated control group for treatment of maxillary deficiency						
Patient or population: Patients in mixed dentition had maxillary deficiency Intervention: Quad–helix Comparison: Untreated control group						
Comments	Certainty of the evidence (GRADE)	No. of participants (studies)	Relative effect (95% CI)	Anticipated absolute effects* (95% CI)		
				Risk after treatment	Risk before treatment	Outcomes
Quad–helix increases the maxillary inter‐molar width compared with control group	⊕⊕⊕◯ Moderate^a^	96 (2 RCTs)	—	MD 4.70 higher (4.20 lower to 5.21 higher)		Maxillary inter‐molar width—RCTs
The evidence suggests that quad–helix increases the maxillary inter‐molar width compared with control group	⊕⊕◯◯ Low^a^^,^ b ^,^ c	124 (2 non ‐RCTs)	—	MD 4.43 higher (3.78 lower to 5.08 higher)		Maxillary inter‐molar width— Non‐RCTs
Quad–helix increases the maxillary inter‐canine width compared with control group	⊕⊕⊕◯ Moderate^a^	96 (2 RCTs)	—	MD 2.47 higher (1.97 lower to 2.96 higher)		Maxillary inter‐canine width—RCTs

^a^ There was very high heterogeneity between the included studies.

^b^ No information on risk of bias for measurements of outcomes in one included study.

^c^ There was high heterogeneity between the included studies.

**Table 5:** Summary of findings according to GRADE.

Removable plate compared before and after for treatment of maxillary deficiency							
Patient or population: Patients in mixed dentition had maxillary deficiency Intervention: Removable plate Comparison: Before and after treatment							
Comments	Certainty of the evidence (GRADE)	No. of participants (studies)	Relative effect (95% CI)	Anticipated absolute effects (95% CI)			
				Risk after treatment	Risk before treatment		Outcomes
The treatment by removable plate increased the maxillary inter‐molar width by 4.05 mm with *I* ^2^ = 0 between the included studies	⊕⊕⊕⊕ High	96 (2 RCTs)	—	MD 4.05 higher (2.98 lower to 5.11 higher)			Maxillary inter‐molar width—RCTs
The evidence suggests that removable plate increased the maxillary inter‐molar width by 3.54 mm with *I* ^2^ = 0 between the included studies	⊕⊕⊕◯ Moderate^a^	556 (3 non ‐RCTs)	—	MD 3.54 higher (3.12 lower to 3.96 higher)			Maxillary inter‐molar width—Non‐RCTs
Removable plate increased the maxillary inter‐canine width by 2.28 mm with *I* ^2^ = 0 between the included studies	⊕⊕⊕⊕ High	96 (2 RCTs)	—	MD 2.28 higher (1.29 lower to 3.27 higher)			Maxillary inter‐canine width—RCTs
The evidence suggests that removable plate increased the maxillary inter‐canine width by 3.71 mm with *I* ^2^ = 0 and without significant heterogeneity between the included studies *I* ^2^ = 0	⊕⊕⊕◯ Moderate^a^	468 (3 non‐RCTs)	—	MD 3.71 higher (3.31 lower to 4.11 higher)			Maxillary inter‐canine width—Non‐RCTs
The evidence suggests that removable plate increased the maxillary width by 1.71 mm without significant heterogeneity between the included studies *I* ^2^ = 0	⊕⊕⊕◯ Moderate^b^	114(2 non ‐RCTs)	—	MD 1.71 higher (0.82 lower to 2.60 higher)			Maxillary width—Non‐RCTs

^a^ No information risk of bias for measurements of outcomes in two included study.

^b^ No information risk of bias for measurements of outcomes in one included study.

**Table 6:** Summary of findings according to GRADE.

Expansion plate compared with untreated control group for treatment of maxillary deficiency						
Patient or population: Patients in mixed dentition had maxillary deficiency Intervention: Expansion plate Comparison: Untreated control group						
Comments	Certainty of the evidence (GRADE)	No. of participants (studies)	Relative effect (95% CI)	Anticipated absolute effects* (95% CI)		
				Risk after treatment	Risk before treatment	Outcomes
The treatment by removable plate increased the maxillary inter‐molar width by 3.71 mm compared with control group	⊕⊕⊕◯ Moderate^a^	96 (2 RCTs)	—	MD 3.71 higher (3.14 lower to 4.28 higher)		Maxillary inter‐molar width—RCTs
Removable plate increased the maxillary inter‐canine width by 2.28 mm with *I* ^2^ = 0 between the included studies compared with control group	⊕⊕⊕⊕ High	96 (2 RCTs)	—	MD 2.28 higher (1.73 lower to 2.83 higher)		Maxillary inter‐canine width—RCTs

^a^ There was a very high heterogeneity between the included studies.

**Table 7:** Summary of findings according to GRADE.

Quad–helix compared with expansion plate for treatment of maxillary deficiency							
Patient or population: Patients in mixed dentition had maxillary deficiency Intervention: Quad–helix Comparison: Expansion plate							
Comments	Certainty of the evidence (GRADE)	No. of participants (studies)	Relative effect (95% CI)	Anticipated absolute effects* (95% CI)			
				Risk after treatment	Risk before treatment		Outcomes
The treatment by quad–helix increased the maxillary inter‐molar width by 1.16 mm without significant heterogeneity between the included studies compared with removable plate	⊕⊕⊕⊕ High	96 (2 RCTs)	—	MD 1.16 higher (0.43 lower to 1.89 higher)			Maxillary inter‐molar width—RCTs
Quad–helix increases maxillary inter‐molar width more than removable plate, but the evidence is uncertain	⊕◯◯◯ Very low^a^^,^ b ^,^ c ^,^ d	61(2 non ‐RCTs)	—	MD 2.35 higher (1.56 lower to 3.15 higher)			Maxillary inter‐molar width—Non‐RCTs
The evidence suggests that quad–helix may increase/decrease the maxillary inter‐canine width slightly compared with expansion plate, and the difference between both groups is non‐significant	⊕⊕◯◯ Low^c^^,^ e	96 (2 RCTs)	—	MD 0.04 higher (−0.67 lower to 0.75 higher)			Maxillary inter‐canine width—RCTs
Quad–helix may increase maxillary inter‐ canine width more than removable plate, but the evidence is uncertain	⊕◯◯◯ Very low^a^^,^ b ^,^ d	53 (2 non‐RCTs)		MD 1.64 higher (0.81 lower to 2.48 higher)			Maxillary inter‐canine width—Non‐RCTs

^a^ No information risk of bias for measurements of outcomes in one included study.

^b^ Moderate risk of bias for deviation from intended intervention and no information risk of bias for measurements of outcomes.

^c^ There was very high heterogeneity between the included studies.

^d^ The number of patients who provide data is low.

^e^ The CI crosses the clinical decision‐making threshold for an acceptable estimate of treatment.

## 4 Discussion

Maxillary arch expansion is a common and essential procedure in orthodontic treatment during the mixed dentition stage (Lanteri et al. 2018). Recently, the method of SME, utilizing various appliances such as the QH and the RP, has become increasingly popular. This approach is primarily used to treat maxillary deficiency and posterior crossbite, as it allows for the bone to adapt and remodel in response to applied forces, reducing the need for tooth extraction or surgical treatments in the future (Bell and LeCompte 1981).

This systematic review was conducted to evaluate the skeletal and dental changes that occur during SME.

Both the QH and the RP significantly expanded in terms of the IC and IM width, as previous studies have shown that when comparing a control group with an experimental group that had been treated with QH (Godoy et al. 2011; Erdinç et al. 1999; Petrén and Bondemark 2008; Shundo et al. 2012) or RP (Godoy et al. 2011; Erdinç et al. 1999; Petrén and Bondemark 2008). Also, when the pre‐ and post‐treatment results were compared for both devices, QH (IM 5.7 mm, IC = 5.17 mm) (Godoy et al. 2011; Erdinç et al. 1999; Vargo et al. 2007; Petrén and Bondemark 2008; Huynh et al. 2009; Wong et al. 2011; Corbridge et al. 2011; Shundo et al. 2012; Shoaib et al. 2017) and RP (IM = 4.65 mm, IC = 3.5 mm) (Godoy et al. 2011; Erdinç et al. 1999; Petrén and Bondemark 2008; Van de Velde et al. 2021; Zhang et al. 2023). These results are consistent with the findings of Inchingolo et al. (2023), who compared fixed expansion appliances (RME, SME) at different ages and found a significant increase in IC and IM width; Caroccia et al. (2020), who assessed the effects of early treatment of unilateral posterior crossbite in primary and early mixed dentition using various expansion methods between 2002 and 2020, but they measured just IM width and revealed a significant increase in it (Caroccia et al. 2020). Finally, Zuccati et al. (2013), who observed significant dental expansion (IM and IC width) across various SME methods, whereas they collected RCTs in the last 12 years that applied RME, SME: QH or RP, or SRME in young and adult patients, and they had assessed dental expansion without skeletal expansion (Zuccati et al. 2013).

In this study when the effectiveness of the QH and RP was compared with each other, it became clear that the QH was more effective in increasing the IM (WMD = 1.71; 95% CI: 1.17–2.25; *p* < 0.002) and IC width(WMD = 0.71; 95% CI: 0.17–1.25; *p* < 0.001) (Godoy et al. 2011; Boysen et al. 1992; Erdinç et al. 1999; Petrén and Bondemark 2008), but only Petren's study has showed greater IC width when they used RP in comparison with using QH, the reason could be attributed to the different activation protocol where it was 0.2 mm once a week in RP and every 6 weeks for QH group (Petrén and Bondemark 2008).

These comparative results are in agreement with Petrén et al. (2003), who assessed the effects of early treatment of unilateral posterior crossbite in primary and early mixed dentition Using various expansion methods and reported that the resulting increase from the QH is greater than that from the RP (QH: 3.3–6.4 mm in the molar region and 1.3–5.2 mm in the canine region; RP: 2.6–4.7 mm in the molar region and 0.7– 4.1 mm in the canine region) (Petrén et al. 2003), and with Aboalnaga (2021) study who has shown high quality evidence supported the dentoalveolar effects of SME in growing patients (Aboalnaga 2021). Also, the finding of QH agrees with the view of Bucci et al. (2016), who reported significant increases in maxillary IM and IC widths with QH in a systematic review or meta‐analysis comparing RME and SME with fixed appliances (Bucci et al. 2016).

Skeletal changes occurred during the QH treatment, and their percentage ranged from 14.7% to 35.84% (Frank and Engel 1982; Boysen et al. 1992; Sandikçiolu and Hazar 1997; Erdinç et al. 1999). However, Shoaib et al. (2017) reported that the percentage of skeletal changes when QH was used was 14.17%, which was due to the difference in measurement methods; thus, MW was measured on frontal cephalometric, and IM width was taken on dental casts (Shoaib et al. 2017). On the other hand, in 2011, Corbridge et al. reported that this ratio was 60% because they measured the MW on the palatal side via axial CBCT, which led to a MW close to the IM width; thus, the ratio MW/IM width was large (Corbridge et al. 2011).

Skeletal changes occurred during treatment by RP; the percentage stretched 19.6%–27.7% (Boysen et al. 1992; Erdinç et al. 1999). However, Defraia et al. (2007) reported an average percentage of 90.68%, which was a high ratio; this can be because the treatment began at the end of the primary dentition stage, the treatment period was 10 months, and the second frontal photo was taken after 2 years (Defraia et al. 2007). Additionally, Zhang et al. (2023) reported that the percentage of skeletal changes was 52.67%, which was caused by the activated protocol (two turns a week), the patients' age was very young, the treatment period was 9.09 ± 4.94 months, and the second CBCT photo was taken after 3 months of retention (Zhang et al. 2023).

It can be noted that dental expansion consistently exceeds skeletal expansion, and the degree of dental expansion was always greater than the skeletal expansion with both appliances (Erdinç et al. 1999; Corbridge et al. 2011; Shoaib et al. 2017; Zhang et al. 2023). It was significantly expanded with a mean of 3.42 mm with QH (Erdinç et al. 1999; Corbridge et al. 2011; Shoaib et al. 2017) and a mean of 1.71 mm with RP (Erdinç et al. 1999; Zhang et al. 2023). But when comparing QH with RP, QH induces greater basal expansion (QH: 1.4 ± 1.2 mm and RP: 0.5 ± 0.6 mm) (Erdinç et al. 1999). This result is in agreement with Bucci et al. (2016) study, who compared RME and SME with fixed appliances and found a significant increase of skeletal transversal dimension in the short‐term, and the skeletal expansion is always smaller than the amount of dentoalveolar expansion (Bucci et al. 2016). Given these advantages, SME using the QH has emerged as a viable alternative to rapid maxillary expansion (RME) in early mixed dentition cases (Lanteri et al. 2018).

In terms of treatment time, one study showed that the QH demonstrates higher success rates and shorter treatment durations compared to the RP (Godoy et al. 2011), the result consistent with Zucca's systematic review, which revealed that the QH appliance was superior to the RP in treatment time (Zuccati et al. 2013).

Publication bias was assessed using funnel plots which indicating no significant asymmetry. Subgroup analyses was performed to explore sources of heterogeneity. These additional analyses strengthen the robustness of the finding.

In conclusion, this systematic review highlights SME as an effective non‐surgical approach for maxillary expansion, with the QH demonstrating superior performance to the RP in most parameters. The evidence confirms that while dental expansion predominates, significant skeletal changes also occur, particularly with the QH appliance. These findings support the use of SME, especially in growing patients, as a conservative alternative to more invasive treatments. Further standardization of measurement protocols could help clarify the variability in reported skeletal expansion percentages across studies.

While this study provides comprehensive evidence on the skeletal and dentoalveolar effects of SME in mixed dentition, several limitations must be acknowledged, including the heterogeneity of measurement methods across studies, with only 4/28 studies using CBCT (Corbridge et al. 2011; Zhang et al. 2023; Brunetto et al. 2013; Serafin et al. 2022) and most relying on dental casts or 2D imaging, the predominance of short‐term outcomes, only two studies followed patients beyond 1 year (Defraia et al. 2007; Zhang et al. 2023), variability in appliance activation protocols, and the lack of age‐stratified analyses despite differences in treatment response between early and late mixed dentition. To address these gaps, future research should prioritize standardized 3D imaging protocols in longitudinal studies with extended follow‐ups, age‐stratified RCTs comparing SME appliances including adjusted force protocols, biomechanical investigations to optimize skeletal adaptation, and patient‐centered outcomes to assess comfort and long‐term stability. In addition, studies should control for confounding factors such as nasal obstruction and mandibular arch dimensions to isolate treatment effects and guide clinical decision‐making.

## 5 Conclusions

SME during the mixed dentition phase can be obtained in varying degrees of skeletal and dentoalveolar alterations. The extent of skeletal changes can range from 16% to 60% of the total changes. The extent depends on the type of expansion device used. Consequently, the QH appliance produces the greatest level of skeletal modifications. As a result, the outcomes achieved from SME encourage us to prefer this method over RME in cases of mild to moderate maxillary compression.

## Author Contributions

S.A.I. conceived, planned, and performed the work leading to the report, interpreted the evidence presented, or both. T.R. wrote the report, reviewed successive versions, approved the final version, and shared in their revisions. M.K. edited the manuscript. M.Y. approved the final version.

## Disclosure

This manuscript is original and is not under consideration by any other journal. All authors have approved this final version and agree with its submission. Registration: PROSPERO Registration CRD42023487507 (12/12/2023).

## Consent

Informed consent was obtained from all subjects.

## Conflicts of Interest

The authors declare no conflicts of interest.

## Supporting information


Supporting File 1

Supporting File 2

## Data Availability

The datasets generated during and/or analyzed during the current study are available from the corresponding author on reasonable request.
