# Supplementary material for: Skeletal Effects of Slow Maxillary Expansion in Mixed Dentition Stage: A Systematic Review and Meta‐Analysis
Source: Clin Exp Dent Res. 2026 Nov 9;12(6):e70488. doi: 10.1002/cre2.70488 (PMC13747248; doi:10.1002/cre2.70488)
Supplement: Supplementary file 1 [file CRE2-12-e70488-s001.docx]

 Funnel plot of comparison: 1 QH treatment, outcome: 1.1 Comparison of QH treatment, outcome: maxillary inter-molar width [mm] without sensitivity analysis (A)

 Funnel plot of comparison: 1 QH treatment, outcome: 1.2 Comparison of QH treatment, outcome: maxillary inter-molar width [mm] with sensitivity analysis (B)

 Funnel plot of comparison: 1 QH treatment, outcome: 1.3 Comparison of QH treatment, outcome: maxillary intercanine width [mm] without sensitivity analysis (A)

 Funnel plot of comparison: 1 QH treatment, outcome: 1.4 Comparison of QH treatment, outcome: maxillary inter-canine width [mm] with sensitivity analysis (B)

 Funnel plot of comparison: 2 RP treatment, outcome: 2.1 Comparison of RP treatment, outcome: maxillary inter-molar width [mm] without sensitivity analysis (A).

 Funnel plot of comparison: 2 RP treatment, outcome: 2.2 Comparison of RP treatment, outcome: maxillary inter-molar width [mm] with sensitivity analysis (B)

 Funnel plot of comparison: 2 RP treatment, outcome: 2.3 Comparison of RP treatment, outcome: maxillary inter-canine width [mm] without sensitivity analysis (A)

 Funnel plot of comparison: 2 RP treatment, outcome: 2.4 Comparison of RP treatment, outcome: maxillary inter-canine width [mm] with sensitivity analysis (B)

 Funnel plot of comparison: 4 QH / C, 3.1 outcome: maxillary inter-canine width [mm] without sensitivity analysis

Funnel plot of comparison: 4 QH / C, outcome: 3.2 comparison between quad-helix and control group, outcome: maxillary inter-canine width [mm] without sensitivity analysis (A) and with sensitivity analysis (B)

Funnel plot of comparison: 4 QH / C, outcome: 3.3 comparison between quad-helix and control group, outcome: maxillary inter-molar width [mm] without sensitivity analysis (A).

Funnel plot of comparison: 4 QH / C, outcome: 3.4 comparison between quad-helix and control group, outcome: maxillary inter-molar width [mm] without sensitivity analysis (A) and with sensitivity analysis (B)

Funnel plot of comparison: 5 RP /C, outcome: 4.1 comparison between removable plate and control group, outcome: maxillary inter-molar width [mm] without sensitivity analysis (A) and with sensitivity analysis (B)

Funnel plot of comparison: 5 RP /C, outcome: 4.2 comparison between removable plate and control group, outcome: maxillary inter-molar width [mm] without sensitivity analysis (A) and with sensitivity analysis (B)

 Funnel plot of comparison: 5 RP /C, outcome: 4.3 comparison between expansion plate and control group, outcome: maxillary intercanine width [mm] without sensitivity analysis (A) and with sensitivity analysis (B)

 Funnel plot of comparison: 5 RP /C, outcome: 4.4 comparison between expansion plate and control group, outcome: maxillary intercanine width [mm] without sensitivity analysis (A) and with sensitivity analysis (B)

 Funnel plot of comparison: 3 QH / RP, outcome: 5.1 comparison between quad-helix and expansion plate, outcome: maxillary inter-molar width [mm] without sensitivity analysis (A) and with sensitivity analysis (B)

 Funnel plot of comparison: 3 QH / RP, outcome: 5.2 comparison between quad-helix and expansion plate, outcome: maxillary inter-molar width [mm] with sensitivity analysis (A) and with sensitivity analysis (B)

 Funnel plot of comparison: 3 QH / RP, outcome: 5.3 comparison between quad-helix and expansion plate, outcome: maxillary inter-canine width [mm] without sensitivity analysis (A) and with sensitivity analysis (B)

 Funnel plot of comparison: 3 QH / RP, outcome: 5.4 comparison between quad-helix and expansion plate, outcome: maxillary inter-canine width [mm] with sensitivity analysis (A) and with sensitivity analysis (B)
