# Supplementary material for: Skeletal Effects of Slow Maxillary Expansion in Mixed Dentition Stage: A Systematic Review and Meta‐Analysis
Source: Clin Exp Dent Res. 2026 Nov 9;12(6):e70488. doi: 10.1002/cre2.70488 (PMC13747248; doi:10.1002/cre2.70488)
Supplement: Supplementary file 2 [file CRE2-12-e70488-s002.docx]

**Table 1: Risk of bias assessment for the included randomized studies using ROB 2-I tool.**

| Study ID | Bias arising from the randomization process | | Bias due to deviation from intended intervention | | Bias due to missing data | | Bias in measurement of outcome | | Reporting bias | | Overall risk judgment |
| --- | --- | --- | --- | --- | --- | --- | --- | --- | --- | --- | --- |
|  | **judgment** | **description** | **judgment** | **description** | **Judgment** | **description** | **judgment** | **description** | **judgment** | **Description** |  |
| Petrén and Bondemark 2008 | **Low** | The patients were randomized into 4 groups.   4 opaque envelopes were prepared with 20 sealed notes in each (5 notes for each group). Thus, for every new patient, a note was extracted from the first envelope. When the envelope was empty, the second envelope was opened, and the 20 new notes were extracted as patients were recruited to the study. This procedure was then repeated 2 more times. The envelope was in the care of 1 investigator, who was contacted and randomly extracted a note and informed the dentist which treatment strategy to use. | **Low** | Blinding of participants and intervention providers is not applicable; outcomes are unlikely to be influenced by this knowledge.  Any deviation from intended intervention reflects usual practice (The dentist thoroughly instructed the patient to use the plate day and night except for meals and tooth brushing) | **low** | Data were available for all participants before and after treatment. | **low** | Outcome measurements were comparable and evaluated by one assessor. The examiner was unaware of which treatment the patients had received or which models were taken at T0 and T1. | **low** | While unclear if authors selected the reported outcome measures a prior, the outcome measures are objective; and all  reported results correspond to all intended outcomes. | **low** |
| Godoy et al., 2011 | **Low** | The 99 children were randomly divided into 3 groups: QDH, expansion plate (EP), and untreated controls. For randomization, numbers were randomly drawn from a plastic bag. Each child received a number from 1 to 99 | **Low** | Blinding of participants and intervention providers is not applicable; outcomes are unlikely to be influenced by this knowledge.  Any deviation from intended intervention reflects usual practice (one orthodontist diagnosed, took impressions,  placed the appliances, and followed the treatment plan). | **low** | Data were available for all participants before and after treatment. | **low** | The investigator was unaware of the type of appliance  used by the patient and the length of treatment | **low** | While unclear if authors selected the reported outcome measures a prior, the outcome measures are objective; and all  reported results correspond to all intended outcomes. | **low** |
| Martina et al., 2012 | **Low** | The study was a randomized controlled trial. Enrolled subjects were allocated to the two treatment groups, that is, RME or SME, by a balanced block randomization using gender as stratifying factor. A single operator (PL) allocated the patients by means of a custom-made Java script and was responsible for the allocation concealment, that is, the allocation was disclosed only when a new patient was enrolled in the trial. | **Low** | All clinical procedures were administered by a single operator (PM), who was not blinded to patient allocation. Any deviation from intended intervention reflects usual practice | **Low** | The data were reasonably complet | **low** | The distances were measured by a single examiner (RN), who was blinded to patient allocation by means of software (Materialise mimics 8.1, Leuven, Belgium). | **low** | The outcome measures are objective; and all  reported results correspond to all intended outcomes | **Low** |
| Brunetto et al., 2013 | **Low** | A sample of 59 subjects was selected and randomly divided into 2 groups: rapid  maxillary expansion and slow maxillary expansion. | **low** | Blinding of participants and intervention providers is not applicable; outcomes are unlikely to be influenced by this knowledge.  Any deviation from intended intervention reflects usual practice | **low** | Data were available for all participants before and after treatment. | **low** | The same operator made all measurements; he was unaware of the group  to which each patient belonged. | **low** | The outcome measures are objective; and all  reported results correspond to all intended outcomes. | **Low** |
| Nagrik et al., 2014 | **Some concerns** | Twenty patients (mean age 11.8 years) with skeletal class II malocclusion, were randomly divided into two groups of 10 each: (low sample size in each group) | **low** | Blinding of participants and intervention providers is not applicable; outcomes are unlikely to be influenced by this knowledge (dental and skeletal changes after treatment). | **low** | Data were available for all participants randomized before and after treatment. | **Some concerns** | Outcome measurements were comparable and evaluated by several investigators  Unclear if outcome assessors were blinded to the intervention status or groups. | **low** | Not enough information to determine whether the reported data were selected; but  All reported results correspond to all intended outcomes | **Some concerns** |
| Paoloni et al., 2021 | **low** | A computer-generated random number list was used to allocate patients to treatments. The allocation sequence was concealed by the statistician (M.N.), who used opaque and sealed envelopes, sequentially numbered for each of the two centers. The envelopes were opened by the operators only when the expander was prepared for cementation | **low** | The clinicians couldn’t be blinded about the treatment that they were providing. The patients and parents did not know the used. However, they could not be blinded, because they could observe the expander that required different activation. The examiner was blinded on the type of expander used. | **low** | Data were available for all participants before and after treatment | **Some concerns** | Outcome measurements were comparable  There were several operators. Unclear if outcome assessors were blinded to the intervention status or groups | **low** | The outcomes mentioned in the protocol have been reported | **Some concerns** |
| Serafin et al., 2022 | **low** | A randomized clinical trial .Every new patient was allocated to the groups using a computer program | **low** | The study was blinded regarding the statistical analysis, eliminating from the elaboration file every reference to patient group assignment | **low** | Data were available for all participants before and after treatment | **low** | Outcome measurements were comparable. Skeletal and dental landmark  were taken using a coordinate system proposed by Lagravère et al., confirmed and measured | **low** | The protocol for this randomized clinical trial was reviewed and approved by the Ethical Committee of the University of Milan | **low** |

**Table 2: Risk of bias assessment for the included non-randomized studies using ROBINS-I tool.**

| Study ID | Pre-intervention | | | | At-intervention | | Post-intervention | | | | | | | | Overall risk judgment |
| --- | --- | --- | --- | --- | --- | --- | --- | --- | --- | --- | --- | --- | --- | --- | --- |
|  | **Cofounding bias** | | **Selection bias** | | **Classification of intervention** | | **Bias due to deviation from intended intervention** | | **Bias due to missing data** | | **Bias in measurement of outcome** | | **Reporting bias** | |  |
|  | **judgment** | **description** | **Judgment** | **description** | **judgment** | **description** | **judgment** | **description** | **Judgment** | **description** | **judgment** | **description** | **judgment** | **Description** |  |
| Shundo et al., 2011 | **Low** | The subjects assigned to the treatment and control groups were  consecutive, nonrandomized patients.  Confounding domains were controlled and matched between groups. | **Low** | Patients were selected retrospectively according to well defined eligibility criteria.  Start of intervention and follow up are coincided. | **Low** | Intervention is well defined. | **low** | Deviations of the intended intervention reflects usual practice | **low** | Data were reasonably complete | **low** | Outcome measurements were comparable. A single investigator  measured the coded cephalograms and dental casts blindly  to group, sex and time of taking them. | **low** | The outcome measures are objective; and all  reported results correspond to all intended outcomes. | **Low** |
| Defraia et al., 2007 | **Moderate** | The treatment group comprised 23 subjects (8 males and 15 females) treated with a removable appliance for the expansion of the maxillary arch. While 20 patients with untreated unilateral posterior crossbite who refused early treatment after the first evaluation were used as the control group.  Some of confounder domains were not measured; however, all pretreatment measurements were matched between the two groups. | **low** | Selection of participant was according to well defined eligibility criteria, and all eligible participant were analyzed.  Start of intervention and follow up are coincided. | **low** | Intervention is well defined and intervention definition is based solely on information collected at the time of intervention. | **low** | Deviations of the intended intervention reflects usual practice | **low** | Data were reasonably complete | **No information** | Outcome measurements were comparable, all tracings were performed by one author and were subsequently verified by another investigator (no information if they were blinded about the exposure status or group); this may have a slight impact on the results | **low** | While unclear if authors selected the reported outcome measures a prior, the outcome measures are objective; and all  reported results correspond to all intended outcomes. | **Moderate** |
| Bell and LeCompte  I981 | **critical** | Prospective comparative study involved only Ten subjects, five in the deciduous dentition and five in the mixed dentition.  Gender distribution and occlusion class were not reported at baseline time.  The effect of normal growth was not considered, which may cause a time varying confounding. | **critical** | Low sample size 10 patients divided into two groups.  Eligibility criteria were not clearly defined.  Start of intervention and follow up were coincided | **low** | Intervention is well defined. | **low** | Deviations of the intended intervention reflects usual practice | **low** | Data were reasonably complete. | **No information** | Outcome were measured by two independent observers. Unclear if they were blinded to the intervention status and the measurement error **was not reported.** | **No information** | Unclear if authors selected the reported outcome measures a prior | **Critical (study was re-evaluated using NIH tool)** |
| Vargo et al., 2007 | **Moderate** | Retrospective study. Historical reference data were used for making untreated comparisons. Z-scores (each patient was matched by age and sex) were calculated to evaluate treatment and post-treatment changes. The Z-score provides a measure of a subject’s arch size in standard deviation units. A significant change in Z-score indicates a treatment effect more or less than expected from normal growth | **Moderate** | 54 records for consecutively treated patients were selected according to well-defined eligibility criteria.  All eligible participants were analyzed.  Follow-up records for evaluating of relapse were obtained only for  patients (n =23) who returned for phase II treatment. However, this may have a slight effect on the results)  Start of intervention and start of follow up were coincided for all patients. | **low** | Intervention is well defined. | **Critical** | Patients in the intervention group were treated in 1 of 2 ways, with a bonded palatal expander or a quad-helix appliance combined with a mandibular banded Crozat/lip bumper, so the treatment effects of each appliance could not be evaluated.  Comparison of normal untreated was based on historical reference data. | **low** | Data regarding pre-post treatment changes were reasonably complete. | **No information** | Outcome measurements were comparable, no information about who performed measurements.  Reliability and Systematic error were assessed by using duplicate measures on a subset of randomly selected models. | **low** | The outcome measures are objective; and all  reported results correspond to all intended outcomes. | **Critical (study was excluded because pre-post treatment comparison for each appliance could not be evaluated)** |
| Frank and Engel 1982 | **Serious** | Uncontrolled retrospective interrupted time series study.  Some of confounder domains were not controlled; e.g. very wide age range in the boys' subgroup (7-17 years).  Effect of growth was predicted using multiple regression analysis. | **low** | Selection of participant was according to well defined eligibility criteria, and all eligible participant were analyzed.  Start of intervention and follow up are coincided. | **low** | Intervention is well defined. | **Low** | The patients had received treatment at two private offices; although treatment protocol was identical for each group, potential bias due to unknown deviation from intended intervention is still remained. However, its impact on the outcome is expected to be slight. | **low** | Data regarding pre-post treatment changes were reasonably complete. | **No information** | Outcome measurements were comparable, no information about who performed measurements.  The measurement error was not reported**.** | **No information** | The outcome measures are objective; and all reported results correspond to all intended outcomes. | **Serious** **(study was reevaluated using NIH tool)** |
| Corbridge et al., 2011 | **Serious** | Retrospective cohort study. The sample consisted of 73 patients (39 boys, 34 girls).  Type of pretreatment malocclusion was not mentioned, which considered an important confounding domain.  Role of natural growth was not considered which may cause time varying confounding. | **Serious** | Patient included if they  have phase 1 quad-helix treatment, a CBCT scan taken before phase 1 (T1), and a CBCT scan taken before phase 2 (T2) treatment; type of constriction or crossbite was not considered in the inclusion criteria.  Patients were treated consecutively. | **Low** | Intervention is well defined | **Low** | Patients were treated by 3 orthodontists although treatment protocol was identical | **Low** | Data regarding pre-post treatment changes were reasonably complete. | **Low** | Outcome measurements were comparable  A total of 146 CBCT scans were evaluated by a blinded examiner  To evaluate reliability, 20 scans were randomly selected, and the measurements were repeated. | **Low** | The outcome measures are objective; and all reported results correspond to all intended outcomes | **Serious (study was reevaluated using NIH tool)** |
| Ciambotti et al., 2001 | **Low** | Prospective comparative study comprised of 12 patients treated with RPE appliances and 13 patients treated with NiTi palatal expansion appliances.  Some of important confounding domains were not controlled as type of crossbite, and age.  However, a stepwise multiple regression analysis was used to evaluate the possible factors related to the overall amount of expansion. | **Serious** | The criteria for patient  selection included patients in mixed or early permanent  dentition who required palatal expansion; type of constriction or crossbite was not considered in the inclusion criteria.  Patients were treated consecutively. | **Low** | Intervention is well defined | **Low** | Deviations of the intended intervention reflects usual practice | **Low** | Data were reasonably complete | **Low** | Outcome measurements were comparable  Four orthodontic faculty members judged the patients’ pretreatment and posttreatment radiographs.  For error measurements, dental casts of 10 patients before and after treatment were used. | **critical** | Treatment changes were reported. However, pre- and post-treatment measurements were not reported | **Critical**  **(study was excluded because pre-post treatment comparison could not be included)** |
| Huynh et al., 2009 | **Low** | Retrospective non-randomized study.  Confounding factors were evaluated and regression models were used to evaluate associations between variables.  Expander type, sex, and retainer use were adjusted to eliminate confounding by these variables.  The treatment effects were separated from growth by comparison with published norms matched with the sex and chronological age of the subjects. | **Low** | All patients were selected according to well defined eligibility criteria.  Subjects were consecutively treated.  Data were collected at the start of treatment (T1), after PXB correction (T2), and at least 2 years posttreatment (T3). | **Low** | Intervention is well defined | **Low** | Patients were treated by one author.  Deviations of the intended intervention reflects usual practice | **Low** | Data were reasonably complete | **Low** | The investigator was blinded with regard  to expander type, sex, and retainer used.  Outcome measurements were comparable and measurement errors were calculated. | **Low** | The outcome measures are objective; and all reported results correspond to all intended outcomes. | **Low** |
| Van de Velde et al., 2021 | **Low** | Prospective cohort study contained 226 patients treated with a removable expansion plate.  Confounding factors were evaluated and linear models were used to assess the association between patient factors (age, gender, crossbite, type of appliance) and the change in canine and molar width. | **Low** | Selection of participant was according to well defined eligibility criteria, and all eligible participant were analyzed.  Start of intervention and follow up are coincided. Records were taken at the start (T0) and at the end of interceptive treatment (T1). | **Low** | Intervention is well defined | **Low** | All patients were treated at the same department of Orthodontics.  Patients were instructed to wear the appliance 24h/day and to remove it only for tooth brushing, rinsing and cleaning.  Deviations of the intended intervention reflects usual practice | **Low** | Data were reasonably complete | **No information** | Outcome measurements were comparable.  The evaluation of the transversal effects was performed by one observer (unclear if he was blinded or not, but this may have a slight impact on the results).  The measurement error was estimated. | **Low** | The outcome measures are objective; and all  reported results correspond to all intended outcomes. | **Low** |
| Shoaib et al., 2017 | **Moderate** | Prospective uncontrolled before-after study consisted of fourteen subjects (7 girls and 7 boys).  outcomes measurements at pre-intervention were reported.  Effect of growth cannot be determined, which may cause a time varying confounding. However, the mean treatment time was 8 months, so effect of growth could be negligible. | **Low** | All patients were selected according to well defined criteria.  Each patient had their panoramic, lateral and posterio-anterior (PA) cephalometric x-rays before and just after the expansion. | **Low** | Intervention is well defined | **Low** | Patients were treated by the same orthodontist and were appointed every week for evaluation. | **Low** | Data were reasonably complete | **Moderate** | Outcome measurements were comparable. All cephalometric radiographs were hand traced by the same examiner.  The measurement error was not reported | **Low** | The outcome measures are objective; and all  reported results correspond to all intended outcomes. | **Moderate** |
| Ferrario et al., 2003 | **Critical** | Low sample size; 13 patients in experimental group were divided into Primary (4 F) and mixed dentition (6 M and 3 F) groups.  7 patients in the Ctrl group were also divided into ‘primary’ (1 M and 1 F) and ‘mixed dentition’ (3 M and 2 F) groups.  During a six-month interval, the natural growth was negligible, as assessed in seven control children. | **Low** | Selection of participant was according to well defined eligibility criteria, and all eligible participant were analyzed.  Start of intervention and follow up are coincided. | **Low** | Intervention is well defined | **Low** | Deviations of the intended intervention reflects usual practice | **Low** | Data were reasonably complete | **No information** | Outcome measurements were comparable  Unclear if the outcome assessors were blinded to the intervention status.  The method error has already been reported. intra-operator repeatability was assessed and for all variables, no systematic errors were found. | **critical** | Only treatment changes were reported. pre- and post-treatment measurements were not reported | **Critical (study was reevaluated using NIH tool)** |
| Bukhari et al., 2018 | **Low** | Retrospective case-control study.  The control and treated samples were matched for gender, molar  relationship, and dental age.  (However, the control group was from another study, so before-after comparison of the treated group was evaluated in this study). | **Low** | Sample was randomly selected according to clear inclusion criteria. | **Low** | The expansion protocol is well defined | **Low** | Deviations of the intended intervention reflects usual practice | **Low** | Data were reasonably complete | **Low** | Outcome measurements were comparable.  The examiner was blinded for crossbite and non-crossbite sides until all data were collected.  Intra-examiner agreement was assessed. | **Low** | The outcome measures are objective; and all  reported results correspond to all intended outcomes. | **Low**  **(For before-after comparison)** |
| Wong Ch et al 2021 | **Low** | Retrospective nonrandomized study; 110 patients were treated by SME using one of the three types of fixed expanders.  Predicted changes for each measurement due to normal growth were calculated via a quadratic interpolation using published norms matched to gender and chronologic age of study subjects. | **Low** | Selection of participant was according to well defined eligibility criteria. Records were taken pretreatment (T1), posttreatment (T2), and early permanent dentition models (T3) | **Low** | Intervention is well defined | **Low** | Deviations of the intended intervention reflects usual practice | **Low** | Data were reasonably complete | **No information** | Measurement techniques were described  Intra-rater reliability was determined.  Unclear if the outcome assessors were blinded to the intervention status | **critical** | All treatment groups were pooled in one group and compared with published matched norms. Therefore, the effects of each appliance could not be determined. | **Critical**  **(Study was excluded because before-after comparison of each treatment group could not be evaluated.** |
| Akkaya et al., 1998 | **Low** | The sample included 24 patients (12 patients in each group) treated either by slow or rapid expansion.  Gender distribution was not reported.  Pre-treatment measurements were reported but only the treatment changes were compared between the two groups.  Therefore, before-after comparison was evaluated in this study. | **Low** | Selection of participant was according to well defined eligibility criteria, and all eligible participant were analyzed.  Start of intervention and follow up are coincided. | **Low** | Intervention is well defined | **Low** | Deviations of the intended intervention reflects usual practice | **Low** | Before, after expansion, and after retention data were complete. | **No information** | Outcome measurements were comparable. No information about who performed measurements  For all casts all parameters were measured again after 15 days and measurement error was determined. | **Low** | The outcome measures are objective; and all reported results correspond to all intended outcomes | **Low**  **(For before-after comparison)** |
| BOYSEN.B et al 1992 | **Low** | The sample for the present study consisted of 34 children (26 girls and eight boys). All children presented a unilateral, symmetrical, posterior crossbite associated with a lateral mandibular shift from centric relation to intercuspal occlusion.  This included several ratios of transverse linear measurements in order to eliminate the effect of craniofacial growth on the enlargement factor. | **Low** | Different records were made for all children of both groups at four different time intervals. | **Low** | Intervention is well defined | **Moderate** | One of the authors (KIC) treated half of the children with a quad-helix and another author treated the other half with a removable Schwarz plate appliance. | **Low** | Data were reasonably complete | **No information** | Outcome measurements were comparable  The measurements were made by one of the authors but unclear if he was blinded or not  The error of method was estimated. | **Low** | The outcome measures are objective; and all reported results correspond to all intended outcomes | **Moderate** |
| Erdinç et al., 1999 | **Low** | Retrospective study with two treatment and control group.  Confounding domains were controlled and matched between groups. | **Low** | Patients were included according to well defined inclusion criteria.  Start of intervention and follow up are coincided. | **Low** | Intervention is well defined | **Low** | Deviations of the intended intervention reflects usual practice | **Low** | Data were reasonably complete | **No information** | The measurements were made by a single investigator but unclear if he was blinded or not.  The measurement error was estimated for all variables. | **Low** | The outcome measures are objective; and all reported results correspond to all intended outcomes | **Low** |
| Sandtkqto~lu and Hazar 1997 | **Serious** | Thirty patients were divided into three groups with 10 patients in each group. The mean ages at the beginning of the treatment for each group were 6.6, 8.6, and 8.9 years, respectively  The wide range between the first group and the others made age varying confounding.  Pre-treatment measurements were not reported. | **Moderate** | Inclusion criteria were unilateral or bilateral posterior crossbites only.  Start of intervention and follow up are coincided. | **Low** | Intervention is well defined | **Serious** | The variance between the treatment times of the semi-rapid group and the slow group can be explained partly with delinquency of parents adjusting the appliances and lack of good cooperation. | **Low** | Data were reasonably complete | **No information** | Outcome measurements were comparable. No information about who performed measurements  the error of the method  was estimated. | **critical** | Pre- and post-treatment measurements were not reported. | **Critical**  **(Study was excluded because pre-post treatment comparison could not be included)** |
| Zhang et al.,2023 | **Low** | Forty four children aged 6–10 years with maxillary transverse defciency who had received ERME treatment in the Department of Pediatric Dentistry, West China Hospital of Stomatology | **Low** | Patients were selected retrospectively according to well defined eligibility criteria.  Start of intervention and follow up are coincided | **Low** | Intervention is well defined | **Low** | Deviations of the intended intervention reflects | **Low** | Data were reasonably complete | **Low** | CBCT images were examined and measured with Mimics software. The lateral cephalometric radiographs were examined and measured with Dolphin Imaging Software | **Low** | The outcome measures are objective; and all reported results correspond to all intended outcomes | **Low** |
| Cossellu et al.,2020 | **Moderate** | Retrospective study with two treatment and control group.  Ninety Caucasian patients, 43 males and 47 females (mean age 7.5 ± 1.5 years), presenting with maxillary transverse deficiency and posterior crossbite. The groups were matched according age, ratio of gender in the same group but there was different in number of patients between groups | **Low** | Selection of participant was according to well defined eligibility criteria | **Low** | Intervention is well defined | **Low** | Digital landmarks were traced by means of 3Shape Ortho Analyzer software (version 1.7). | **Low** | Data were reasonably complete | **Low** | Each patient was randomly identified with a number and eight linear variables were considered, traced and analyzed by a single blinded examiner | **Low** | The study was approved by the Ethic Committee, Fondazione IRCCS Ospedale Maggiore-Policlinico, Milan | **Moderate** |
| Ladner et al.,1995 | **Low** | Retrospective nonrandomized study; 60 patients were treated by SME/RME using fixed expanders  Expander type, sex, and retainer use were adjusted to eliminate confounding by these variables. | **Low** | Selection of participant was according to well defined eligibility criteria | **Low** | Intervention is well defined | **Low** | Deviations of the intended intervention reflects | **Low** | Data were reasonably complete | **No information** | Outcome measurements were comparable. Unclear if the outcome assessors were blinded to the intervention status.  Measurement error was determined. | **Low** | The outcome measures are objective; and all reported results correspond to all intended outcomes | **Low**  **(For pre-post comparison)** |
| Mossaz-Joelson et al.,1989 | **Serious** | The sample consisted of 10 patients, 6 boys and 4 girls, presenting either a bilateral or a functional unilateral crossbite. They were divided into two experimental groups. In group 1 the age ranged between 8 years 7 months and 10 years 9 months. In group 2 it varied from 8 years 8 months to 12 years  (Different in the range of ages between groups and low sample size) | **Low** | Selection of participant was according to well defined eligibility criteria | **Low** | Intervention is well defined | **Low** | Deviations of the intended intervention reflects | **Low** | Data were reasonably complete | **No information** | Outcome measurements were comparable. No information about who performed measurements | **Low** | The outcome measures are objective; and all reported results correspond to all intended outcomes | **Serious**  **(study was reevaluated using NIH tool)** |

**Table 3: risk of bias assessment using NIH quality assessment tool for before-after (Pre-Post) study with no control group**

https://www.nhlbi.nih.gov/health-topics/study-quality-assessment-tools

|  | **Frank and Engel 1982** | **Corbridge et al., 2011** | Mossaz-Joelson et al.,1989 |
| --- | --- | --- | --- |
| **1. Was the study question or objective clearly stated?** | **Yes** | **Yes** | **Yes** |
| **2. Were eligibility/selection criteria for the study population prespecified and clearly described?** | **Yes** | **Yes** | **Yes** |
| **3. Were the participants in the study representative of those who would be eligible for the test/service/intervention in the general or clinical population of interest?** | **Yes** | **Yes** | **Yes** |
| **4. Were all eligible participants that met the prespecified entry criteria enrolled?** | **Yes** | **Yes** | **Yes** |
| **5. Was the sample size sufficiently large to provide confidence in the findings?** | **Yes** | **Yes** | **No** |
| **6. Was the test/service/intervention clearly described and delivered consistently across the study population?** | **Yes** | **Yes** | **Yes** |
| **7. Were the outcome measures prespecified, clearly defined, valid, reliable, and assessed consistently across all study participants?** | **Yes** | **Yes** | **Yes** |
| **8. Were the people assessing the outcomes blinded to the participants' exposures/interventions?** | **NR** | **Yes** | **NR** |
| **9. Was the loss to follow-up after baseline 20% or less? Were those lost to follow-up accounted for in the analysis?** | **Yes** | **Yes** | **Yes** |
| **10. Did the statistical methods examine changes in outcome measures from before to after the intervention? Were statistical tests done that provided p values for the pre-to-post changes?** | **Yes** | **Yes** | **NO** |
| **11. Were outcome measures of interest taken multiple times before the intervention and multiple times after the intervention (i.e., did they use an interrupted time-series design)?** | **Yes** | **Yes** | **NO** |
| **12. If the intervention was conducted at a group level (e.g., a whole hospital, a community, etc.) did the statistical analysis take into account the use of individual-level data to determine effects at the group level?** | **NA** | **NA** | **NA** |
| **Quality rating** | **Good quality** | **Good quality** | **Poor quality** |
| **Additional comments** | The actual changes during each stage of therapy were compared with the normal growth changes that would occur in a statistically equivalent group | Blinding of assessors has a slight impact on outcomes. | Small sample size  Methods to evaluate the measurement error were not reported |

  *CD, cannot determine; NA, not applicable; NR, not reported
